# TRF2–RAP1 represses RAD51-dependent homology-directed telomere repair by promoting BLM-mediated D-loop unwinding and inhibiting BLM–DNA2-dependent 5′-end resection

**DOI:** 10.1093/nar/gkae642

**Published:** 2024-07-31

**Authors:** Fengshan Liang, Rekha Rai, Tori Sodeinde, Sandy Chang

**Affiliations:** Departments of Laboratory Medicine, Yale University School of Medicine, 330 Cedar St., New Haven, CT 06520, USA; Departments of Laboratory Medicine, Yale University School of Medicine, 330 Cedar St., New Haven, CT 06520, USA; Departments of Laboratory Medicine, Yale University School of Medicine, 330 Cedar St., New Haven, CT 06520, USA; Departments of Laboratory Medicine, Yale University School of Medicine, 330 Cedar St., New Haven, CT 06520, USA; Pathology, Yale University School of Medicine, 330 Cedar St., New Haven, CT 06520, USA; Molecular Biophysics and Biochemistry, Yale University School of Medicine, 330 Cedar St., New Haven, CT 06520, USA

## Abstract

Inappropriate homology-directed repair (HDR) of telomeres results in catastrophic telomere loss and aberrant chromosome fusions, leading to genome instability. We have previously shown that the TRF2–RAP1 heterodimer protects telomeres from engaging in aberrant telomere HDR. Cells lacking the basic domain of TRF2 and functional RAP1 display HDR-mediated telomere clustering, resulting in the formation of ultrabright telomeres (UTs) and massive chromosome fusions. Using purified proteins, we uncover three distinct molecular pathways that the TRF2–RAP1 heterodimer utilizes to protect telomeres from engaging in aberrant HDR. We show mechanistically that TRF2–RAP1 inhibits RAD51-initiated telomeric D-loop formation. Both the TRF2 basic domain and RAP1-binding to TRF2 are required to block RAD51-mediated homology search. TRF2 recruits the BLM helicase to telomeres through its TRFH domain to promote BLM-mediated unwinding of telomere D-loops. In addition, TRF2–RAP1 inhibits BLM–DNA2-mediated 5′ telomere end resection, preventing the generation of 3′ single-stranded telomere overhangs necessary for RAD51-dependent HDR. Importantly, cells expressing BLM mutants unable to interact with TRF2 accumulate telomere D-loops and UTs. Our findings uncover distinct molecular mechanisms coordinated by TRF2–RAP1 to protect telomeres from engaging in aberrant HDR.

## Graphical Abstract

**Graphical Abstract:**
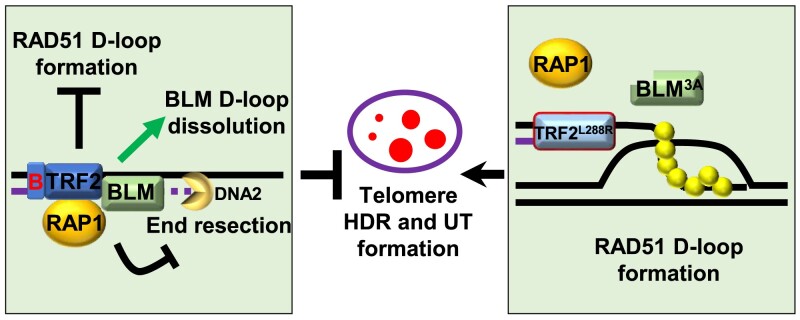

## Introduction

As essential DNA–protein structures at the end of linear eukaryotic chromosomes, telomeres protect chromosomal ends from being recognized as double strand breaks (DSBs) which would otherwise initiate inappropriate DNA damage repair pathways (1,2). The shelterin complex contains six telomere-specific proteins crucial to maintain telomere homeostasis (3,4). Three telomeric repeat-binding factors, TRF1 and TRF2–RAP1, bind telomeric dsDNA, while protection of telomeres 1 (POT1) interacts with TPP1 to bind to the ends of 3′ single stranded (ss) DNA overhang and to the ssDNA–dsDNA junction (3,5). TRF1 and TRF2–RAP1 interact with POT1–TPP1 through another core component of the complex, TIN2 (6). TRF2 catalyzes the formation of the telomere (T)-loop, a structure which sequesters the 3′-G rich overhang into telomeric dsDNA, leading to repression of the ATM-dependent DNA damage signaling pathway and classical (C)-NHEJ-mediated repair (7–11).

The conserved repressor/activator protein 1 (RAP1) forms a heterodimer with TRF2 to protect telomeres from activating ATR-dependent homology-directed repair (HDR) (12–16). RAP1 promotes the affinity and selectivity of the TRF2 basic domain (TRF2^B^) to branched telomere DNA structures, and this interaction safeguards telomere integrity by repressing PARP1 activation and telomere cleavage (17–19). Inappropriate HDR at telomeres result in catastrophic telomere loss and end-to-end chromosome fusions (2,12,14). Previously, we have shown that RAP1 cooperates with TRF2^B^ to impede the formation of telomere-free chromosome fusions by repressing the localization of HDR factors, including PARP1 and SLX4, to telomeres (14). TRF2–RAP1 also contributes to nuclear lamin A assembly to prevent telomere-telomere clustering and the formation of RAD51-dependent ultrabright telomeres (UTs) (15).

During HDR, the RAD51 recombinase forms nucleoprotein filaments on ssDNA, which in turn captures double-stranded (ds) DNA to search for homologous sequences. This results in the formation of the RAD51–ssDNA–dsDNA complex, leading to ssDNA strand invasion and D-loop formation (20–22). Depletion of the HDR tumor suppressor BRCA2 or RAD51 results in telomere shortening and fragility (23), suggesting that HDR factors play important roles in mediating telomere end protection. Both TRF2 and RAD51 possess telomere DNA strand invasion activities (24–26). TRF2 (but not TRF1) is able to inhibit RAD51-mediated telomere D-loop formation and is required to promote BLM helicase-mediated unwinding of telomere DNA substrates (24,27). Both RAD51 and BLM localize on telomeres, and BLM physically interacts with TRF2 in cells that maintain telomere length using the alternative lengthening of telomeres (ALT) mechanism (27–31). These results suggest a potential regulation of telomere HDR by TRF2 through its associations with RAD51 and BLM.

Using highly purified proteins, we now demonstrate that TRF2 cooperates with RAP1 to inhibit RAD51-mediated telomere D-loop formation. Mechanistically, both TRF2^B^ and TRF2′s interaction with RAP1 are required to block RAD51-mediated homology search on telomeres. We show that BLM interacts with TRF2 via a conserved TRFH-binding motif in the BLM helicase domain, and this interaction enhances the ability of BLM to unwind telomeric D-loops. Cells expressing BLM mutants compromised in their interaction with TRF2 accumulate telomere D-loops and form UTs. Finally, we show that TRF2–RAP1 inhibits BLM–DNA2 nuclease-mediated telomere 5′ end resection and the generation of 3′ ssDNA overhangs, an early step in Rad51-mediated HDR. Our results reveal mechanistically that TRF2–RAP1 protects telomeres from engaging in telomere HDR by coordinating at least three distinct pathways to prevent telomere D-loop formation.

## Materials and methods

### Protein expression and purification

#### Mutant construction

All TRF2, RAP1 and BLM point mutations were generated with a site-directed mutagenesis kit (Agilent), and mutant proteins were expressed and purified following the same procedures developed for their wild type counterparts. Cell lysate preparation and all the purification steps were carried out at 4°C.

### TRF2

WT hTRF2 and TRF2^ΔB^ in pTricHisB vectors (Addgene #50 488 and #53 208) were transformed into *Escherichia coli* BL21(DE3) cells. Overnight cultures in 20 ml LB were diluted 40 times with fresh growth medium and incubated at 37°C until the OD_600_ reached 0.6. IPTG was added to 0.4 mM final concentration to induce protein expression, and cells were harvested by centrifugation. 4 g cell pellet was resuspended in 20 ml cell breaking buffer [20 mM Tris–HCl, pH 7.5, 10% sucrose, 0.5 mM EDTA, 300 mM KCl, 0.1% Igepal CA-630, 1 mM DTT, 1 mM PSMF, and a cocktail of protease inhibitors (Roche) and subject to sonication to prepare crude lysate. After centrifugation (14 000 × g, 60 min), the clarified lysate was gently incubated with 2 ml Ni Sepharose 6 Fast Flow (GE healthcare) for 2 h. The beads were sequentially washed twice with 50 ml of buffer K (20 mM Tris–HCl, pH 7.5, 10% glycerol, 0.5 mM EDTA, 0.01% Igepal, 1 mM DTT) containing 1000 mM KCl or 300 mM KCl plus 10 mM imidazole. TRF2 proteins were eluted from the affinity matrix with 1 ml of buffer K containing 500 mM imidazole and 300 mM KCl three times. The eluate was concentrated in a Centricon-30K device (Millipore) to 5 mg/ml and stored in small aliquots at −80°C.

### RAP1

hRAP1 cDNA with a C-terminal Flag-tag was subcloned into the BglII/HindIII site of pTric-HisB vector. hRAP1 protein was expressed in BL21 (DE3) *E. coli* cells and purified by Ni Sepharose affinity chromatography similar to TRF2 purification. The Ni Sepharose eluate was then mixed with anti-FLAG M2 Affinity Agarose Gel (Sigma) for 2 h, followed by protein elution in elution buffer K with 0.3 mg/ml 1 × Flag peptide (Sigma). The eluate was concentrated to 5 mg/ml before being frozen and stored in small aliquots at −80°C.

### BLM

Human BLM cDNA with a C-terminal Flag-tag from pcDNA3/BLM (Addgene #111 766) was subcloned into phCMV1-2 × MBP-MCS vector (32). The phCMV1 construct with 2 × MBP-tagged hBLM were transiently transfected using the calcium phosphate method into 293T cells. All the purification steps were carried out using a protocol previously described (33) with modifications below. In brief, the cells were harvested 32 h post-transfection and lysed in buffer A (25 mM Tris–HCl, pH 7.5, 10% sucrose, 0.5 mM EDTA, 300 mM KCl, 1% Igepal CA-630, 1 mM DTT, 1 mM PSMF and a cocktail of protease inhibitors). The lysate was cleared by ultracentrifugation for 20 min at 10 000 g, and the supernatant was batch bound to amylose resin (New England Biolabs) overnight. The proteins were then eluted with buffer K with 300 mM KCl and 50 mM maltose. The eluate was then mixed with anti-FLAG M2 Affinity Agarose Gel for 2 hrs, followed by protein elution in elution buffer K with 300 mM KCl and 0.3 mg/ml 1× Flag peptide. The eluates were concentrated to 500 ng/μl with a 2 ml 100 kDa Centrifugal Filter (Amicon) and stored in 2 μl aliquots at −80°C.

### Other recombination proteins

Purification of human RAD51 was performed using published procedures (34,35).

### DNA substrates

The sequences of all oligonucleotides used in this study are listed in Supplementary Table S1. The telomere DNA oligo (T100) was used in the D-loop assay. The PBB plasmid containing telomere sequence (TTAGGG)_17_ was purchased from Addgene (#53 210). The non-telomere DNA oligo (90mer) and plasmid for D-loop assay were described previously (35,36). 5′-end biotin labeled telomere oligo (T100) and non-telomere oligo (90mer) were used in the RAD51/ssDNA filament stabilization and dsDNA capture assays. The dsDNA containing telomere sequence (TTAGGG)_17_ used for DNA binding or dsDNA capture assay was a DNA fragment isolated from PBB using BamHI/HindIII. The non-telomere dsDNA was generated by NonTDC1 and NonTDC2 annealing. The telomere D-loop substrate was generated by hybridizing 5′ ^32^P-labeled oligonucleotide TD2 to oligonucleotides TD1 and TD3. The oligos to generate the non-telomeric D-loop structure are NonTD1, NonTD2 (5′ ^32^P-labeled) and NonTD3. The oligos T3O1 and T3O2 (5′ ^32^P-labeled) were used to generate the telomere 3′-overhang. The oligos to form the non-telomere 3′-overhang (NonT3O1 and 5′ ^32^P-labeled NonT3O2) were described previously (37).

### Telomere DNA binding assay

Purified proteins (25–400 nM) were incubated with 5′ ^32^P radiolabeled telomeric dsDNA (30 nM) in 10 μl reaction buffer D [25 mM Tris–HCl (pH 7.5), 50 mM KCl, 1 mM DTT, 100 mg/ml BSA, and 1.5 mM MgCl_2_] at 37°C for 10 min. The reaction mixtures were resolved in 10% polyacrylamide gels in 1 × Tris-borate-EDTA (TBE) buffer [40 mM Tris–HCl (pH 8.3), 45 mM boric acid, and 1 mM EDTA] at 4°C. After drying, the radiolabeled DNA species were visualized by phosphorimaging.

### Protein affinity pull-down assay

Flag-tagged RAP1 (2 μg) and His-tagged TRF2 (2 μg) were incubated in 30 μl reaction buffer P [25 mM Tris–HCl (pH 7.5), 10% glycerol, 0.5 mM EDTA, 0.01% Igepal, 1 mM DTT, and 150 mM KCl] on ice for 30 min, and then 15 μl anti-Flag M2 affinity resin was added to capture RAP1 through its Flag-tag. After gentle mixing at 4°C for 1 h, the resin was washed three times with 30 μl of buffer P and then treated with 30 μl of 2% SDS to elute bound proteins. The supernatant and SDS eluate (10 μl each) were analyzed by 7.5% SDS-PAGE and Western blotting with antibodies against TRF2 or Flag (for RAP1). For affinity pull-down reactions involving His-tagged TRF2 and untagged RAD51 or MBP-tagged BLM, Nickel NTA resin or amylose resin was used to capture the tagged proteins. The supernatant and eluates from the affinity resin were analyzed by SDS-PAGE and Coomassie blue staining.

### Nuclear extract preparation and D-loop assay

Nuclear extracts (NEs) were generated as previously described (25). Nuclear extract (50 μg) was incubated with 5 ng ^32^P -labeled telomere ssDNA (T100, Supplementary Table S1) in 20 μl buffer E (10 mM HEPES, pH 7.4, 10 mM KOAc, 1 mM ATP, 5 mM CaCl_2_, 1 mM MgCl_2_, 1 mM DTT and proteases inhibitors) and incubated at 37°C for 10 min. 5 μg of PBB plasmid was then added and incubated at 37°C for 30 min. The reaction mixtures were deproteinized by treatment with 5% SDS and 8 mg/ml proteinase K at 37°C for 10 min before being subjected to 1% agarose gel electrophoresis. Gels were dried and analyzed by phosphorimaging as previously described (35).

### RAD51-mediated telomere D-loop assay

The assay was conducted as described previously (35,36) with modifications. Briefly, ^32^P-labeled telomere ssDNA (T100) (2.5 μM) and RAD51(0.8 μM) were pre-incubated in 10.5 μl of reaction buffer A (40 mM Tris, pH 7.5, 1 mM DTT, 1 mM MgCl_2_, 5 mM CaCl_2_ and 50 mM KCl containing 2 mM ATP) at 37°C for 10 min. TRF2 and/or RAP1 at various concentrations (200, 400, 800 nM) was pre-incubated with plasmid PBB containing telomere sequence (TTAGGG)_17_ (35 μM) on ice for 10 min. RAD51/ssDNA filaments generated were mixed with TRF2–RAP1/plasmid and incubated for 25 min at 37°C. The reaction was deproteinized by proteinase K and SDS followed by 1% agarose gel electrophoresis and phosphorimaging analysis. For the non-telomere D-loop assay, the oligo 90mer and pBlueScript replicative form I DNA (35) were used.

### RAD51-telomere ssDNA filament stabilization assay

This assay was conducted at 37°C, and reaction mixtures were resolved by electrophoresis in 10% polyacrylamide gels as described previously (38) with modifications. Briefly, 4 μl of streptavidin resin immobilized with 5′-biotinylated oligo T100 (10 μM) was incubated with RAD51 (2.7 μM) in 18 μl of reaction buffer (40 mM Tris–HCl, pH 7.5, 50 mM KCl, 2 mM ATP, 1 mM MgCl_2_, 5 mM CaCl_2_, 100 μg/ml BSA, and 1 mM DTT) containing an ATP-regenerating system consisting of 20 μM creatine phosphate and 20 μg/ml creatine kinase for 10 min at 37°C. TRF2 and/or RAP1 (200 or 400 nM) was added and incubated at 37°C for 20 min. Then, as a protein trap, excess un-biotinylated oligo T100 (100 μM) in 1 μl was added in the reaction, and the reaction mixed gently for 10 min at 37°C. The resin was centrifuged at 2000 rpm for 2 min and washed twice with 20 μl of reaction buffer containing 0.01% Igepal and 1 mM DTT, and the bound proteins were eluted with 20 μl of 2% SDS. Gels in which the supernatant fraction and the SDS eluate have been resolved were Coomassie blue stained to determine RAD51 protein content.

### Telomere duplex DNA capture assay

This assay was conducted at 37°C and reaction mixtures were resolved by electrophoresis in 10% polyacrylamide gels as described previously (35) with modifications. Briefly, streptavidin resin immobilized with 5′-biotinylated oligo T100 (10 μM) was incubated with RAD51 (3 μM) in 20 μl of reaction buffer W (35 mM Tris–HCl, pH 7.5, 50 mM KCl, 2 mM ATP, 1 mM MgCl_2_, 5 mM CaCl_2_,100 μg/ml BSA and 1 mM DTT) for 10 min. The resin was centrifuged at 2000 rpm for 2 min and washed once with 20 μl buffer and resuspended in 19 μl buffer W. TRF2 and RAP1 either individually or in combination (200 or 400 nM) were pre-incubated with ^32^P radiolabeled telomere dsDNA (4 μM) on ice for 10min. The TRF2/RAP1/dsDNA mixture was added to the resin and incubated at 37°C for 20 min. The resin was centrifuged and washed twice with 20 μl buffer W, and the bound proteins and radiolabeled DNA were eluted with 20 μl of 2% SDS. Gels in which the supernatant and SDS eluate have been resolved were dried and subject to phosphorimaging analysis to reveal and quantify the radiolabeled dsDNA. For the non-telomere duplex DNA capture assay, 5′-biotinylated oligo 90mer (10 μM) and ^32^P radiolabeled dsDNA (oligonucleotides NonTDC1/NonTDC2) were used.

### D-loop dissolution and 3′- end overhang DNA unwinding assay

To generate oligo-based telomere D-loop substrate, ^32^P-labeled oligonucleotide TD2 was hybridized with oligonucleotides TD1 and TD3. TRF2, RAP1 or the combination of both proteins (40 or 80 nM) were pre-incubated with the D-loop DNA (2.5 nM) on ice for 10 min. Then BLM (20 nM) was added and incubated in buffer M containing an ATP-regenerating system at 37°C for 20 min. Reaction mixtures were deproteinized and analyzed by electrophoresis in 10% native polyacrylamide gels.

RAD51, ^32^P-labeled telomeric ssDNA (T100) and plasmid PBB were used to generate telomere D-loop DNA. The deproteinized reaction mixtures by SDS and proteinase K were passed through Micro Bio-Spin 6 Column (Bio-Rad), equilibrated with buffer M (20 mM Na-HEPES [pH 7.5], 2 mM ATP, 0.1 mM DTT, 100 mg/ml BSA, 0.05% Triton X-100, 2 mM MgCl_2_ and 100 mM KCl) and an ATP-regenerating system consisting of 20 mM creatine phosphate and 30 μg/ml creatine kinase. TRF2 and RAP1 either individually (40 or 80 nM) or in combination were pre-incubated with the RAD51-generated D-loop DNA (2.5 nM) on ice for 10 min. Then BLM (20 nM) was added and incubated at 37°C for 20 min. Reaction mixtures were deproteinized and analyzed as described in the D-loop assay.

Telomere 3′ G-overhang DNA was created by annealing ^32^P-labeled oligonucleotide T3O2 with oligonucleotide T3O1. TRF2, RAP1 individually or in combination were pre-incubated with the 3′ G-overhang DNA (5 nM) on ice for 10 min. Then BLM (20 nM) was added and incubated at 37°C for 20 min. Reaction mixtures were deproteinized and analyzed on native PAGE gel. For non-telomere D-loop DNA substrate generation, ^32^P-labeled oligonucleotide NonTD2 was hybridized with oligonucleotides NonTD1 and NonTD3. To create non-telomere 3′-overhang DNA substrate, ^32^P-labeled oligonucleotide NonT3O2 was hybridized with NonT3O1.

### DNA end resection assay

TRF2, RAP1 (40 or 80 nM) individually or in combination were pre-incubated with telomere 3′-end overhang DNA (1 nM) on ice for 10min. Then BLM (10 nM)/RPA (100 nM) /DNA2 (10 nM) was added and incubated in buffer M containing an ATP-regenerating system at 37°C for 20 min. The reactions were then incubated for 5 min at 37°C after adding SDS (0.2%) and proteinase K (0.25 mg/ml). Assay products were separated on 10% nondenaturing polyacrylamide gels in TBE buffer. Gels were dried and subject to phosphorimaging analysis.

### Co-immunoprecipitation and western blotting

U2OS cells were co-transfected with pcDNA3 containing Flag-tagged cDNA of the WT or mutant forms of BLM and with pcDNA3 containing Myc-tagged TRF2 cDNA. Following PBS buffer wash, whole cell lysates were prepared by adding 500 μl lysis buffer (50 mM Tris–HCl, pH 7.7, 150 mM NaCl, 0.5% NP-40, 1 mM DTT and protease inhibitors). After sonication for 10 s on ice, the cell lysates were cleared by centrifugation (14 000 × g, 15 min) at 4°C. The protein concentrations were determined using the Bradford assay (Bio-Rad) and 2 mg of protein was adjusted to 1 ml using lysis buffer, and then incubated with 50 μl anti-FLAG M2 affinity resin (Sigma) with gentle rocking at 4°C for 16 h. Resins were washed 5 times with 500 μl ice cold lysis buffer and then treated with 2% SDS to elute bound proteins. The following antibodies were used in western blotting: α-Flag (Millipore), α-Myc (Fisher).

### Cell culture and transfection

The generation and cell culture of shTRF2 and TRF2 mutants were described previously (15). The 3′-UTR shBLM with target sequence 5′- GAATCTCAATGTACATAGA −3′ was cloned into the pSUPER.retro.puro vector (Clontech). 293T cells were transfected with the shRNA by LipoD293 (SignaGen) to generate shBLM retroviral supernatants. HA-tagged cDNAs coding for the WT and mutant forms of BLM were introduced into the pQCXIP vector (Clontech). Transient transfections of HA-BLM and BLM mutants were performed using Fugene 6. DNA constructs for sh*TRF2*, sh*BLM* and Myc-TRF2^ΔB; L288R^ were transfected into 293 T cells using Fugene 6 and packaged into retroviral particles. Viral supernatants were collected 48–72 h after transfection, filtered with 0.45 μm Millex filter and directly used to infect U2OS cells.

### Immunofluorescence microscopy and fluorescent *in situ* hybridization

Cells grown on coverslips were fixed for 10 min in 2% (w/v) sucrose and 2% (v/v) paraformaldehyde at RT followed by PBS washes. Coverslips were blocked in 0.2% (w/v) fish gelatin and 0.5% (w/v) BSA in PBS. Cells were incubated with primary antibodies and after PBS washes, cells were incubated with appropriate Alexa fluor secondary antibodies followed by washes in PBS + 0.1% Triton. For IF-FISH, the cells were further fixed with 4% (w/v) paraformaldehyde for 10 min, followed by hybridization with TelC-Cy3 (CCCTAA)_3_ PNA telomere probe (PNA Bio, F1002) in hybridization buffer (0.5 μg/ml tRNA, 1 mg/ml BSA, 0.06 × SSC, 70% formamide), denatured at 85 °C for 3 min and then incubated at RT overnight in a humid chamber. After washing the coverslips, DNA was stained with DAPI (Vectashield # H1200), and digital images captured at 10 or 100 ms exposure using NIS-Elements BR (Nikon) with a Nikon Eclipse 80i microscope and an Andor CCD camera.

### Statistical analyses

In biochemistry assays, band intensity was quantified using Quantity One (Bio-Rad). Statistical analysis was conducted by GraphPad Prism 9 software. Statistical differences were determined by one-way ANOVA or unpaired *t* test. ns: non-significant (*P* ≥ 0.05); *: 0.01 < *P*< 0.05; **:0.001 < *P*< 0.01; ***: 0.0001 < *P*< 0.001; ****: *P*< 0.0001.

## Results

### The TRF2 basic domain cooperates with RAP1 to inhibit RAD51-mediated telomere D-loop formation

**Figure 1.**
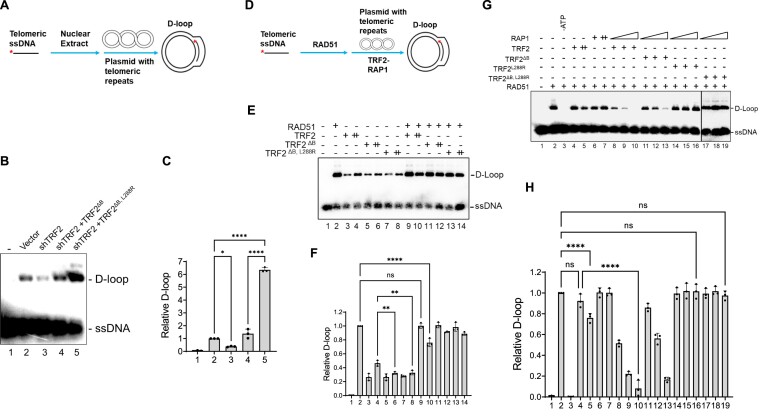
TRF2–RAP1 inhibits RAD51-mediated telomere D-loop formation. (**A**) Schematic of the nuclear extract D-loop assay. ^32^ P-labeled telomere ssDNA was incubated with nuclear extract isolated from cells and telomere plasmids containing (TTAGGG)_17_ repeats. Successful invasion of the ssDNA into the plasmid forms the telomere D-loop structure. Asterisk (*) denotes the ^32^P-labeled ssDNA end. (**B**) Cells expressing the TRF2^ΔB, L288R^ mutant accumulate RAD51-induced D-loops on telomeres. Nuclear extract from U2OS cells expressing empty vector, shTRF2, and shTRF2-depleted cells expressing TRF2^ΔB^ or TRF2^ΔB, L288R^ mutants were examined for telomere D-loop formation. The ssDNA and generated D-loops were separated on 1% agarose gel. (**C**) Quantification of telomere D-loop formation described in (B). D-loops relative to empty vectors (lane 2) from three independent experiments were shown as the mean ± S.D. Statistical evaluation was performed via one-way ANOVA. *P= 0.0126; ****P< 0.0001. (**D**) Schematic of the RAD51 or/and TRF2-mediated telomere D-loop assay. ^32^P-labeled telomere ssDNA was first incubated with purified RAD51 protein to form RAD51–ssDNA filament, and then mixed with TRF2 and/or RAP1-preincubated telomere plasmid. The RAD51–ssDNA strand invasion into the protein-bound plasmid formed telomere D-loops. Asterisk indicates the labeled ssDNA end. (**E**) TRF2 only slightly inhibited RAD51-mediated telomere D-loop formation. RAD51, TRF2, TRF2^ΔB^, or TRF2 ^ΔB, L288R^ and the indicated protein combinations were tested for their ability to form telomere D-loops. (**F**) D-loop formation relative to control (RAD51 alone, line 2) from three independent experiments were analyzed as shown in (E). Mean values ± S.D. were plotted. Data were evaluated by one-way ANOVA analysis. ‘ns’ indicates no significant differences (*P* > 0.05); **P= 0.0033 and 0.0057; ****P< 0.0001. (**G**) TRF2 and RAP1 synergized to promote RAD51-dependent telomere D-loop inhibition. TRF2, RAP1 and the indicated protein combinations (400 nM of TRF2 and 200, 400, 800 nM of RAP1) were tested for their ability to affect RAD51-mediated telomere D-loop formation. The reaction without ATP was used as negative control (lane 3). (**H**) Quantification of D-loops generated in Figure 1G compared to RAD51 alone (lane 2) are analyzed. Data from three independent experiments were shown as the mean ± S.D. in the bottom panel. Statistical evaluation was performed by one-way ANOVA analysis. ns, non-significant (*P* > 0.05); ****P< 0.0001.

We have previously shown that TRF2–RAP1 is required to protect telomeres from HDR-mediated end-to-end chromosomal fusions (14). Expression of a mutant TRF2 lacking the basic domain and RAP1-binding ability (TRF2^ΔB, L288R^) produced RAD51-dependent UTs, since knockdown of RAD51 significantly reduced UT formation. This result suggests that aberrant telomere HDR is responsible for UT formation (15). Previous results suggest that both RAD51 and TRF2 possess the ability to form telomere D-loops (24,25). We hypothesized that UTs form as a result of telomere-telomere HDR (2). To understand mechanistically how TRF2 and RAP1 participate in UT formation, we developed a telomere D-loop assay that mimics the steps required for telomere HDR (25). We incubated nuclear extracts (NE) isolated from U2OS cells expressing empty vector, shTRF2, and cells expressing both shTRF2 and TRF2^ΔB^ or TRF2^ΔB, L288R^ shRNA resistant mutants with ^32^P-labeled telomeric ssDNA and a plasmid containing (TTAGGG)_17_ telomere repeats (Figure 1A). NE from U2OS cells expressing empty vector readily formed telomere D-loops (Figure 1B, lane 2). Inactivation of endogenous TRF2 by shRNA reduced D-loop formation (Figure 1B, lane 3), confirming a role for TRF2 in generating telomere D-loops (24,25). TRF2^B^ plays a role in repressing telomeric D-loop formation, since reconstituting shTRF2-depleted NE with TRF2^ΔB^ increased slightly the level of telomere D-loops formed (Figure 1B, compare lanes 2 and 4). Importantly, NE from cells expressing TRF2^ΔB, L288R^ increased telomeric D-loop formation by ∼6-fold compared to vector control (Figure 1B, lanes 2 and 5 and Figure 1C, Supplementary Figure S1A). This result suggests that both TRF2^B^ and RAP1 are required to repress generation of telomere D-loops, in agreement with our previous data showing that TRF2^B^ and RAP1 repress UT formation (15). Increased D-loops observed in cells expressing TRF2^ΔB, L288R^ is dependent upon endogenous RAD51, since knockdown of RAD51 dramatically decreased telomere D-loops in cells expressing TRF2^ΔB, L288R^ (Supplementary Figures S1B(i) and B(ii)).

To understand mechanistically how TRF2^B^ and RAP1 function to repress RAD51-mediated telomere D-loop formation, we expressed full-length human TRF2, RAP1 or RAD51 in *E. coli* and purified these proteins to near homogeneity (Supplementary Figure S1C). Protein affinity pull-down assays in the presence of 150 mM KCl verified that TRF2 and RAP1 interact directly and that mutant TRF2^ΔB^ binds RAP1 as well as wild type TRF2 (Supplementary Figure S1D(i), lanes 4 and 8). Both TRF2–RAP1 and TRF2^ΔB^ -RAP1 heterodimers remain stable even at 300 mM KCl (Supplementary Figure S1D(ii)). Functional assays revealed that while purified TRF2 and TRF2^ΔB^ efficiently formed heterodimers with purified RAP1, the TRF2^L288R^ and RAP1^F336R^ mutant proteins known to disrupt TRF2–RAP1 binding do not form heterodimers (Supplementary Figure S1D) (13,15). We used purified proteins to perform *in vitro* D-loop assays (Figure 1D) to determine how TRF2 and RAP1 regulate RAD51-mediated D-loop formation on telomeres (35,39). RAD51 alone displayed the recombinase activity to efficiently form ATP-dependent telomere D-loops (Figures 1E, lane 2; 1G, lanes 2 and 3; Figure 1F). TRF2 inhibited the ability of RAD51 to generate D-loops only at high concentrations, and this inhibition requires its basic domain (Figure 1E, compare lanes 10 and 12; Figure 1F) while RAP1 alone was unable to inhibit RAD51-mediated telomere D-loop formation (Figure 1G, compare lanes 2 and 7). However, the combination of WT TRF2 and RAP1 significantly inhibited the ability of RAD51 to form telomere D-loops by several folds compared to RAD51 alone (Figure 1G, compare lane 2 with lanes 8–10). We found that RAP1 has no effect on either wild type or mutant TRF2′s ability to form telomere D-loops in the absence of RAD51 (Supplementary Figure S1E(i) and E(ii)). This result suggests that the inhibition of RAD51-mediated telomere D-loop formation by TRF2–RAP1 is not due to RAP1′s effect on TRF2. Efficient inhibition of RAD51-mediated D-loop formation by TRF2–RAP1 requires an intact TRF2 basic domain (Figure 1G, compare lanes 8–10 with 11–13). Given that the TRF2^ΔB^-RAP1 complex remains as stable as WT proteins even at 300 mM KCl (Supplementary Figure S1D(ii)), the reduced activity of TRF2^ΔB^-RAP1 in suppressing RAD51-mediated D-loop formation is not due to reduced stability of the TRF2^ΔB^-RAP1 complex. We show that the TRF2 basic domain is required for TRF2′s interaction with RAD51, and that TRF2^ΔB^–RAP1 does not interact with RAD51(Supplementary Figures S1F(i), S1F(ii)). These results indicate that efficient inhibition of RAD51-mediated D-loop formation by TRF2–RAP1 requires an intact TRF2 basic domain.

We next asked whether the enhanced inhibition of telomere D-loop formation by the combination of TRF2 and RAP1 is contingent upon heterodimer formation. The TRF2^L288R^ mutant cannot interact with RAP1 and was also unable to inhibit RAD51-mediated telomere D-loop formation (Figure 1G, lanes 14–19). The TRF2^ΔB^ mutant, while still retaining the ability to bind RAP1 (Supplementary Figures S1D(i) and D(ii)), displayed partial inhibition of RAD51′s ability to form telomere D-loops (Figure 1G, lanes 11–13). Inhibition of RAD51 by TRF2–RAP1 was specific to telomere DNA, since the non-telomere D-loops catalyzed by RAD51 were unaffected by the presence of TRF2 or the TRF2–RAP1 complex (Supplementary Figures S1G(i) and G(ii)). Thus, our *in vitro* assays reveal that both TRF2^B^ and RAP1–TRF2 interaction are required to inhibit the generation of RAD51-mediated telomere D-loops.

### TRF2–RAP1 blocks RAD51-ssDNA capture of telomere dsDNA

**Figure 2.**
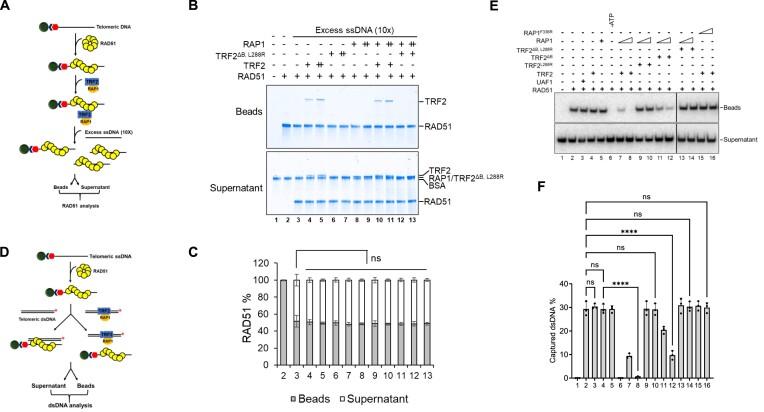
TRF2–RAP1 blocks RAD51-mediated homology search on telomeres. (**A**) Schematic of the assay used to examine the effect of TRF2–RAP1 on the stability of RAD51-coated telomere ssDNA filaments. RAD51 protein was used to assemble filaments on 5′-biotinylated telomere ssDNA that was immobilized on streptavidin resin. TRF2 or/and RAP1 was incubated with the RAD51 coated telomere ssDNA filaments and then 10 × excess of non-biotinylated telomere ssDNA was added in the reaction. RAD51 protein levels in both the beads and the supernatant fractions were analyzed by SDS-PAGE and Coomassie Blue-staining. (**B**) TRF2–RAP1 does not affect RAD51 filament stabilization on telomere ssDNA. TRF2 and RAP1(200 or 400 nM) either individually or in combination were tested for RAD51 telomere ssDNA filament stabilization. The bead fractions containing proteins associated with the biotinylated ssDNA and the supernatant fractions containing proteins trapped on the excess ssDNA were analyzed by SDS-PAGE with Coomassie Blue staining. (**C**) The percentage of RAD51 protein in the supernatant and bead fractions were plotted as mean ± S.D. from two independent experiments. Statistical evaluation was performed by unpaired t test analysis. ns, non-significant (*P*> 0.05). (**D**) Schematic of the telomere dsDNA capture assay by the RAD51 filament. RAD51 was used to assemble filaments on 5′-biotinylated telomere ssDNA that was immobilized on streptavidin resin, and then these filaments were incubated with ^32^P-labled telomere dsDNA either alone or bound to TRF2–RAP1. Radiolabeled dsDNA on the bead and supernatant fractions were resolved by electrophoresis in 10% native polyacrylamide gels. (**E**) TRF2–RAP1 inhibits the ability of RAD51-bound telomere ssDNA filaments to capture telomere dsDNA. TRF2, RAP1 (200 or 400 nM) either singly or in combination were incubated with RAD51 coated telomere ssDNA filaments, and their ability to capture telomere dsDNA was analyzed. ATP was omitted from the reaction in lane 6. DNA-binding protein UAF1 was used as a negative control (lane 3). (**F**) The percentages of captured telomere dsDNA in the bead fractions are shown. The error bars represent mean values ± S.D. of data from three independent experiments. Statistical evaluation was performed by ANOVA. ns: non-significant (*P*> 0.05); ****P< 0.0001.

RAD51–ssDNA nucleoprotein filament formation is a prerequisite to form D-loops. We asked if TRF2–RAP1 affects the stabilization of RAD51 filament on telomere ssDNA, using a biotin-tagged telomere ssDNA assay that enabled DNA pull-down using streptavidin (SA) coated beads (Figure 2A). We first assembled RAD51 on telomere ssDNA and then challenged this complex with 10× excess of telomere ssDNA. In the absence of excess ssDNA, RAD51 stably bound telomere ssDNA and localized to the bead fraction (Figure 2B, lane 2). Approximately 50% of RAD51 was found in the supernatant in the presence of excess telomere ssDNA (Figure 2B, lane 3; Figure 2C). Under these conditions, the addition of purified TRF2 or RAP1 alone or in combination had no effect on RAD51 filament stabilization (Figure 2B, lanes 4–13). These results suggest that the addition of TRF2 or RAP1 does not impact stably formed RAD51–telomere ssDNA complexes.

The stable RAD51–ssDNA nucleoprotein filament possesses the ability to capture duplex DNA to perform homology search, leading to assembly of RAD51–ssDNA–dsDNA complex and ssDNA strand invasion to form D-loops (20–22). We next tested whether RAD51 bound telomere ssDNA filaments still retained the ability to capture TRF2 bound telomere dsDNA. We pre-incubated biotin-labeled telomere ssDNA with RAD51, then introduced ^32^P-labeled telomere dsDNA or telomere dsDNA bound with TRF2–RAP1. SA-coated beads then pulled down the biotin-tagged telomere ssDNA (Figure 2D). As expected, the RAD51–telomere ssDNA filament displayed ATP-dependent dsDNA capture activity (Figure 2E, lanes 2 and 6; Figure 2F). The additional TRF2 or RAP1 alone had no significant effect on this reaction (Figure 2E, lanes 4 and 5). In contrast, the addition of the TRF2–RAP1 heterodimer complexed with telomere dsDNA significantly blocked the ability of RAD51-telomere ssDNA filament to capture telomere dsDNA (Figure 2E, lanes 7 and 8). Both TRF2^L288R^ and RAP1^F336R^ mutants defective in forming TRF2–RAP1 heterodimers were ineffective in blocking RAD51–ssDNA binding to dsDNA (Figure 2E, lanes 9, 10, 13–16). In this regard, the TRF2^ΔB^ mutant also showed partial defect in blocking RAD51-ssDNA binding to dsDNA (Figure 2E, lanes 11 and 12). Importantly, the DNA-binding protein UAF1 (40) possessing similar telomere dsDNA binding activities as TRF2 (Supplementary Figure S2A) does not inhibit the RAD51-ssDNA invasion activity (Figure 2E, lane 3). These results suggest that the inhibition of RAD51–ssDNA mediated capture of telomere dsDNA by TRF2–RAP1 is not due simply to protein coating on the telomere dsDNA substrate. We confirmed that the TRF2–RAP1 complex inhibits RAD51-mediated telomere duplex capture in an ATP-dependent manner (Supplementary Figure S2B). TRF2–RAP1 inhibition of dsDNA capture by RAD51-ssDNA is specific only to telomeres, since this heterodimer has no effect on RAD51-mediated non-telomere dsDNA capture (Supplementary Figure S2C). Thus, the TRF2–RAP1 heterodimer is required to block RAD51-mediated homology search on telomeres.

### TRF2–RAP1 promotes BLM-mediated unwinding of telomere D-loops

**Figure 3.**
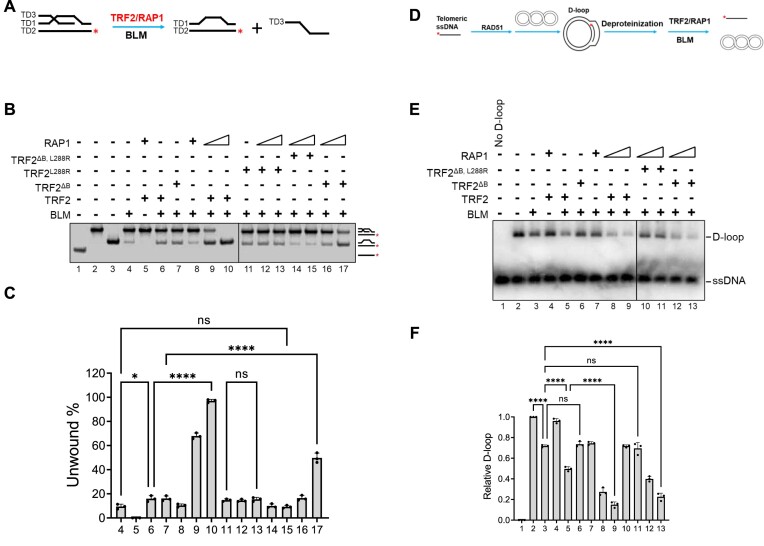
TRF2–RAP1 promotes BLM-mediated unwinding of telomere D-loops. (**A**) Schematic of the oligo-based telomere D-loop unwinding assay. Telomere D-loop DNA substrates were generated by hybridizing three telomere DNA fragments (TD1, ^32^P-labeled TD2 and TD3). TRF2 and/or RAP1 were pre-incubated with the D-loop and then BLM was added to the reaction to detect D-loop unwinding. Displacement of the invading telomere DNA strand from the D-loop indicates D-loop unwinding. (**B**) The TRF2–RAP1 promotes BLM-mediated unwinding of telomere D-loops. The effect of TRF2 and indicated mutants either singly or in combination with RAP1 on BLM-mediated telomere D-loop unwinding was examined. The sizes of ^32^P-labled telomere ssDNA, D-loops, and dsDNA without the invading telomere strand are shown in lanes 1–3. (**C**) Quantification of BLM-mediated unwinding reactions displayed in Figure 3B. The percentages of unwound D-loop are shown as mean values ± S.D. from three independent experiments. Statistical evaluation was performed by ANOVA test. ns: non-significant (*P*> 0.05); *P= 0.0186; ****P< 0.0001. (**D**) Schematic of the assay used to measure the ability of BLM to unwind RAD51-generated telomeric D-loops. Telomeric D-loops were generated by incubating RAD51, ^32^P-labled telomeric ssDNA and plasmid containing telomere repeats together as described in Figure 1D. Native plasmid-sized telomere D-loops were obtained after deproteinization and gel purification. BLM without or with TRF2–RAP1 was then incubated with these D-loops and their unwinding was analyzed by 1% agarose gel. (**E**) BLM-mediated unwinding of RAD51-genearted telomeric D-loops was enhanced by TRF2–RAP1. WT TRF2 or various TRF2 mutants complexed with RAP1 were incubated with D-loops and tested for their effects on the ability of BLM to unwind D-loops. (**F**) The amount of D-loops formed relative to the negative control (no protein, lane 2) were quantified and plotted as mean ± S.D. from three independent experiments. Statistical evaluation was performed by ANOVA test. ns indicates non-significant (*P*> 0.05); ****P< 0.0001.

BLM, a RecQ-like helicase, possesses both pro- and anti-recombinogenic activities, including the ability to disrupt RAD51-ssDNA filament formation and stimulation of DNA repair synthesis to maintain genome stability (41,42). Cellular and biochemical evidence suggest a role for the BLM helicase in telomere maintenance. BLM-mediated unwinding of telomere substrates is enhanced by TRF2 (27–30). To determine whether the TRF2–RAP1 complex is required to promote BLM-mediated telomere DNA unwinding, we purified the BLM protein and developed a telomere D-loop unwinding assay. An oligo-based telomere D-loop structure was generated by annealing an invading DNA oligonucleotide containing telomere TTAGGG repeats into a partial dsDNA paired with G and C-rich strands. TRF2–RAP1 was pre-incubated with D-loops and then BLM was added to detect D-loop unwinding. Displacement of the invading strand from D-loops indicates unwinding (Figure 3A). Consistent with previous findings (27), TRF2 enhanced BLM’s ability to unwind telomere D-loops (Figure 3B, compare lanes 4 and 6; Supplementary Figure S3, lanes 3–7; Figure 3C). While RAP1 alone does not impact BLM activity, the TRF2–RAP1 complex significantly increased the ability of BLM to unwind telomere D-loops (Figure 3B, compare lanes 4, 8 with 9 and 10; Supplementary Figure S3). The TRF2^ΔB^-RAP1 heterodimer only partially promoted BLM’s unwinding activity. However, both TRF2^L288R^ and TRF2^ΔB, L288R^ mutants unable to interact with RAP1 were significantly less effective on BLM’s unwinding activity than the WT TRF2–RAP1 complex (Figure 3B, lanes 12–17). These results suggest that both TRF2^B^ and RAP1′s interaction with TRF2 are required to enhance BLM-mediated telomere D-loop unwinding.

We next used the RAD51-generated telomere D-loop assay (Figure 3D) to show that while BLM alone is able to unwind telomere D-loops, this activity is increased in the presence of WT TRF2 (Figures 3E, compare lane 2 with lane 5; Figure 3F). Importantly, in contrast with the oligo-based D-loop unwinding assay (Figure 3B, lanes 6 and 7), TRF2^ΔB^ mutant failed to promote BLM unwinding on the RAD51-generated D-loops (Figure 3E, compare lanes 5 and 6). TRF2–RAP1, but not the TRF2^ΔB, L288R^ mutant, significantly enhanced BLM’s D-loop unwinding activity (Figures 3E, compare lanes 8, 9 with 10, 11; Figure 3F). Taken together, our results indicate that TRF2–RAP1 promotes BLM-mediated unwinding of telomere D-loops.

### TRF2 interacts with BLM to promote BLM unwinding activity on telomere D-loops

**Figure 4.**
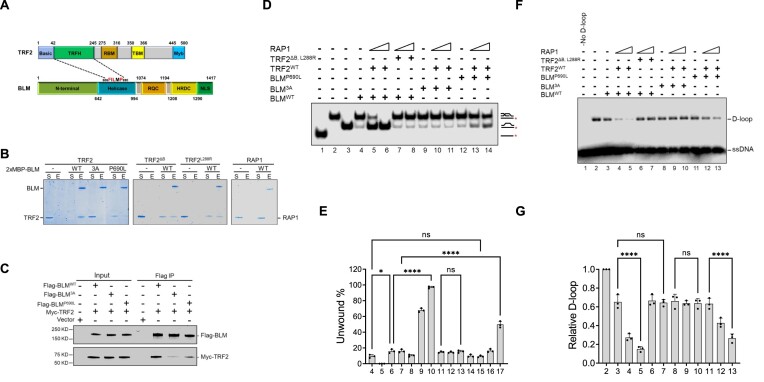
TRF2–BLM interaction is required to promote BLM-mediated unwinding of telomere D-loops. (**A**) Diagram showing that the TRF2 TRFH domain interacts with specific residues in BLM helicase domain containing the conserved TRFH binding motif FILMP (amino acids 686–690). Other functional domains in TRF2 are also illustrated: N-terminal basic domain, RAP1-binding motif, TIN2-binding motif and C-terminal Myb domain. The BLM N-terminal domain, winged-helix motif (RQC); helicase/RNase D C-terminal domains (HRDC) and nuclear targeting signal domain are also illustrated. (**B**) BLM pulldown using its Maltose Binding Protein tag by amylose-resin revealed robust interaction between BLM and TRF2. His-tagged TRF2 and its mutants (TRF2^ΔB^ or TRF2^L288R^) and Flag-tagged RAP1 were incubated with MBP-tagged BLM and BLM mutants (3A or P690L). The protein complexes were captured on amylose-resin and the fractions were resolved on SDS-PAGE gel and visualized with Coomassie Blue staining. S, supernatant with unbound proteins; E, SDS eluate of the amylose-resin. (**C**) Co-IP analysis was used to examine BLM interaction with TRF2 in U2OS cells. Lysates from cells expressing WT Flag-BLM, mutant Flag-BLM (3A or P690L) and Myc-TRF2 were subject to co-IP analysis using anti-FLAG M2 agarose resin. Proteins were detected by Western blotting using anti-Flag or anti-Myc antibodies. (**D**) TRF2–RAP1 does not promote unwinding of telomere-D-loops by BLM mutants exhibiting reduced binding to TRF2. The effect of TRF2 and RAP1 on the ability of WT and mutant BLM (3A or P690L) to unwind telomeric D-loops were tested as in Figure 3B. ^32^P-labled ssDNA, D-loop, and dsDNA without the invading strand were loaded as size markers (lanes 1–3). (**E**) The percentages of unwound D-loops are shown as mean ± S.D. from three independent experiments. Statistical difference was evaluated via ANOVA test. Not significant: *P*> 0.05; ****P< 0.0001. (**F**) TRF2–RAP1 failed to promote BLM^3A^ mutant on unwinding of RAD51-generated D-loops. The effects of TRF2–RAP1 on BLM wild type or 3A, P690L mutants on D-loop unwinding were tested as in Figure 3E. (**G**) The relative D-loop to the control without protein (lane 2) in the bottom panel is shown as mean ± S.D. from three independent experiments. ANOVA test was used to evaluate statistical difference. ns: non-significant (*P*> 0.05); ****P < 0.0001.

BLM and TRF2 co-localize on ALT-associated PML bodies, and BLM’s C-terminus and helicase domains interact with TRF2 (27). However, specific amino acids required for this interaction are not known. We have previously shown that the TRF2 TRFH domain interacts with telomere-associated proteins to promote telomere end protection (14,43). We analyzed the sequence of BLM helicase domain and found that it harbors a 686-FILMP-690 sequence, which is a canonical TRF2 TRFH-binding motif (Figure 4A) (44). To ascertain the functional significance of BLM’s FILMP sequence, we mutated residues F686, L688 and P690 into alanines to generate the BLM^3A^ mutant. Because expression of a BLM single nucleotide polymorphism (P690L) results in hypersensitivity to hydroxyurea and an increased frequency of sister chromatid exchanges (45,46), we also generated a BLM^P690L^ mutant. We purified WT BLM, BLM^3A^ and BLM^P690L^ proteins to near homogeneity (Supplementary Figure S4A) to test their interaction with WT TRF2. Using a protein affinity pulldown assay, we found that both BLM^3A^ and BLM^P690L^ displayed reduced interaction with TRF2 (Figure 4B). In agreement with these results, co-immunoprecipitation with Flag-tagged BLM and Myc-tagged TRF2 revealed that while WT BLM interacted robustly with TRF2, both BLM^3A^ and BLM^P690L^ mutants displayed markedly reduced interactions with TRF2 (Figure 4C). Both BLM mutants retain the activity to co-IP TRF1, revealing that the interaction with TRF2 is indeed specific to the TRF2^TRFH^ (Supplementary Figure S4B). In addition, both BLM mutants display telomere D-loop unwinding activities as robustly as WT BLM (Supplementary Figure S4C). Our data reveal that the BLM mutants are specifically compromised in their interaction with TRF2.

We next asked whether the enhancement of BLM-mediated telomere D-loop unwinding by TRF2–RAP1 is dependent on BLM’s interaction with TRF2. On non-telomere D-loop substrates, the TRF2–RAP1 heterodimer was unable to promote BLM unwinding nor impact D-loop formation (Supplementary Figure S4D, E). The telomere D-loop assay revealed that while TRF2–RAP1 enhanced telomere D-loop unwinding by WT BLM, D-loop unwinding was compromised in the presence of BLM^3A^ and BLM^P690L^ mutants (Figure 4D, E, compare lanes 4–6 with 9–11 and 12–14). Finally, although the BLM^3A^ mutant retains its helicase activity to unwind RAD51-generated telomere D-loops, this unwinding activity was not enhanced by TRF2–RAP1 (Figures 4F, G). Taken together, our data reveal that interaction of TRF2^TRFH^ with BLM is required to enhance BLM-mediated unwinding of telomere D-loops.

### TRF2–RAP1 promotes BLM helicase activity on the telomere 3′- overhang

**Figure 5.**
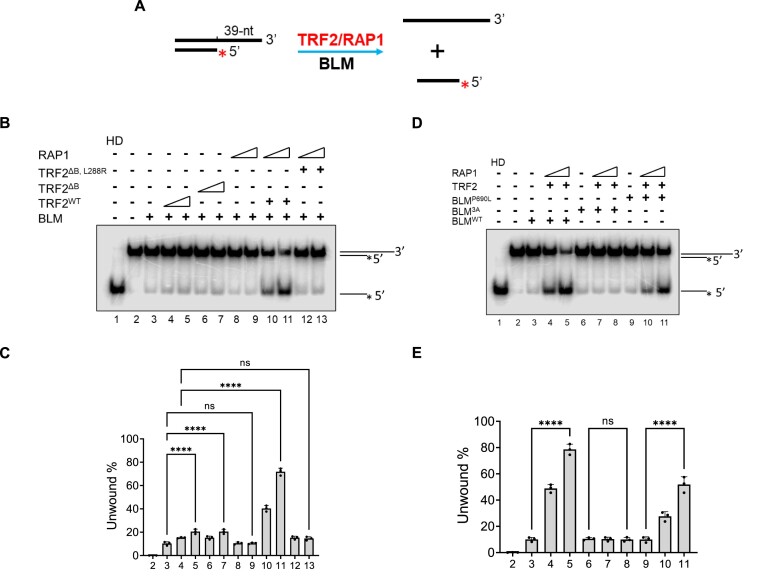
Both RAP1 and BLM binding to TRF2 are required to promote BLM helicase unwinding activity on the telomere 3′ overhang. (**A**) Schematic of the telomere 3′ overhang unwinding assay. A telomere dsDNA with a 39-nt 3′ overhang (GGGTTA)_6_GGG was generated by annealing a telomeric ssDNA containing (TTAGGG)_8_ repeats (T3O1) with another shorter telomere ssDNA oligo containing (TAACCC)_2_ repeats (T3O2). TRF2–RAP1 was first pre-incubated with the telomere dsDNA substrate and then BLM was added to detect unwinding of the 3′ overhang. (**B**) TRF2–RAP1 promotes BLM unwinding on the telomere 3′-overhang. The effect of TRF2 either alone or in combination with RAP1 on the ability of BLM to unwind the telomere 3′-overhang was tested. Heat denatured (HD) ^32^P-labled telomere ssDNA (lane 1) and 3′-overhang dsDNA (lane 2) were loaded as markers. (**C**) Quantification of unwound telomere 3′-overhangs shown in Figure 5B. The percentages of unwound telomere 3′-overhang are shown as mean ± S.D. from three independent experiments. ANOVA test was used to evaluate statistical difference. non-significant: *P*> 0.05; ****P< 0.0001. (**D**) TRF2-BLM binding is required to promote BLM-mediated unwinding of the telomere 3′-overhang. The effect of TRF2–RAP1 on the ability of WT BLM, BLM^3A^ or BLM^P690L^ mutants to unwind telomere 3′-overhangs were tested as in Figure 5B. (**E**) Quantification of BLM-mediated unwinding reactions shown in Figure 5D. The percentages of unwound telomere 3′-overhang from three independent experiments are shown as mean ± S.D. Statistical evaluation was performed by ANOVA test. non-significant: *P*> 0.05; ****P< 0.0001.

Formation of the G-rich 3′-telomere single-stranded overhang is required for the proper maintenance and protection of telomeres (47,48). We utilized a double-stranded telomere substrate bearing a 3′ ss G-overhang (termed T-overhang) to determine whether TRF2–RAP1 plays a role in BLM-mediated unwinding of telomere DNA substrates containing 3′ overhangs (27,37). Telomere dsDNA containing 3′ overhangs (GGGTTA)_6_GGG were generated by annealing telomere ssDNA containing (TTAGGG)_8_ repeats with another shorter telomere DNA oligo containing (TAACCC)_2_ repeats. TRF2–RAP1 was pre-incubated with the DNA substrate and then BLM was added to detect unwinding of the 3′ overhang (Figure 5A). BLM alone was able to unwind the T-overhang, and TRF2 alone slightly promoted this unwinding activity (Figure 5B, compare lanes 3 and 5; Figure 5C). While RAP1 alone had no effect on BLM-mediated unwinding of T-overhangs, the addition of both RAP1 and TRF2 enhanced BLM-mediated T-overhang unwinding several folds over TRF2 alone (Figure 5B, compare lanes 10, 11 with lanes 4, 5). Robust interaction between BLM and TRF2 is required for BLM to unwind T-overhangs, since the unwinding ability of BLM^3A^ was not enhanced by TRF2–RAP1 (Figure 5D, lanes 7, 8). In support of this observation, TRF2–RAP1 was only able to partially enhance BLM^P690L^-mediated T-overhang unwinding (Figure 5D, compare lanes 10, 11 with lanes 4, 5). TRF2–RAP1 played no role in enhancing BLM-mediated unwinding of a non-telomere 3′ overhang substrate (Supplementary Figure S5A, B). These results reveal that TRF2–RAP1 is required to promote BLM-mediated unwinding of telomere 3′ overhangs.

### TRF2–RAP1 inhibits BLM–DNA2-mediated DNA end resection

**Figure 6.**
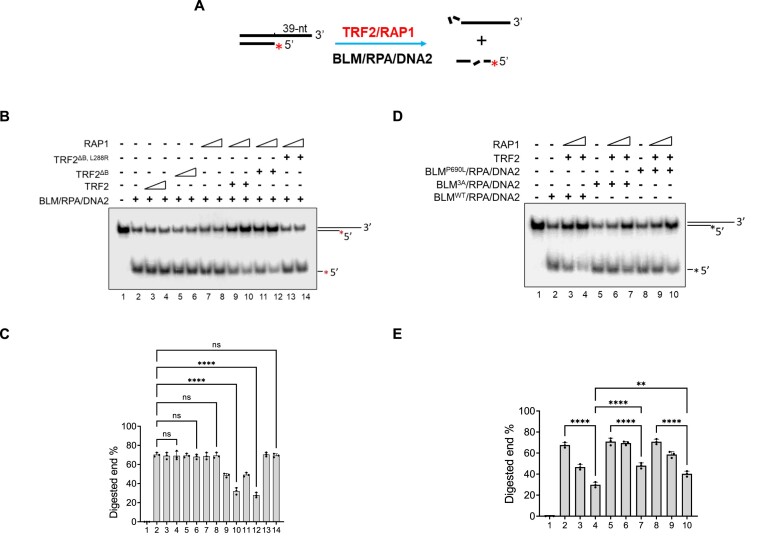
TRF2–RAP1 inhibits BLM–DNA2-mediated telomere 5′ end resection. (**A**) Schematic of the telomere 3′ overhang end resection assay. Telomere dsDNA with a 39-nt 3′overhang was pre-incubated with TRF2–RAP1 and then BLM/RPA/DNA2 added to examine telomere dsDNA end resection. (**B**) TRF2–RAP1 inhibits BLM–DNA2-mediated telomere DNA end resection. WT TRF2, TRF2 mutants (TRF2^ΔB^ or TRF2^ΔB, L288R^) and RAP1 individually or in combination were pre-incubated with telomere dsDNA containing a 3′-overhang. DNA end resection was examined after the BLM/RPA/DNA2 mixture were added to the reaction. (**C**) Quantification of telomere DNA end resection in Figure 6B. The percentages of digested 5′-labeled telomere ssDNA were plotted. The error bars represent mean values ± S.D. of data from three independent experiments. Statistical evaluation was performed by ANOVA test. Non-significant: *P*> 0.05; ****P< 0.0001. (**D**) TRF2 binding to BLM contributes to the inhibition of telomere dsDNA end resection. WT BLM or BLM mutants (3A and P690L) were incubated with RPA and DNA2. The effects of TRF2–RAP1 on the end resection were examined as in Figure 6B. (**E**) Quantification of percentage inhibition of telomeric end resection examined in Figure 6D. The percentages of digested telomere 5′-ends are shown as mean ± S.D. from three independent experiments. Statistical analysis was performed by ANOVA. **P= 0.0016; ****P< 0.0001.

The length of telomere 3′-single-stranded overhangs must be tightly regulated to prevent the formation of abnormally long telomere ssDNA which would otherwise serve as substrates for aberrant HDR (47). In classic DSB repair, long-range 5′-end resection is catalyzed by the exonuclease Exo1 or by the endonuclease DNA2 together with BLM (49–52). DNA2 has been shown to associate with telomeres and its deficiency results in telomere loss (53). We asked whether TRF2–RAP1 plays a role in the regulation of 3′ overhang length by modulating the 5′ telomere end resection activity of DNA2. The telomere dsDNA substrate with 39-nt 3′overhangs (Figure 5A) was pre-incubated with TRF2–RAP1 (Figure 6A). Since BLM is the helicase required to separate dsDNA strands to generate 5′-DNA flaps and RPA stimulates 5′-end cleavage by DNA2 (54), these proteins were also included in the reaction. We found that the DNA2-RPA-BLM complex induced unwinding and 5′-end resection of the T-overhang substrate (Figure 6B, lane 2; Figure 6C). While the addition of TRF2 or RAP1 alone had no detectable impact on this reaction, the combination of both TRF2 and RAP1 significantly inhibited 5′-end resection of the T-overhang (Figure 6B, lanes 9, 10; Supplementary Figure S6A). Interaction of BLM with TRF2 played only a minor role in 5′-end resection, since both BLM^3A^ and BLM^P690L^ mutants displayed the same 5′ end-resection activity as WT BLM (Figure 6D, lanes 2, 5, 8; Figure 6E). In addition, TRF2^B^ does not appear to play a role in inhibiting telomere 5′-end resection (Figure 6B, lanes 9–12). Importantly, TRF2–RAP1 has no effect on BLM–DNA2-mediated 5′ end resection of a non-telomere substrate (Supplementary Figure S6B). Our results suggest that TRF2–RAP1 inhibits BLM–DNA2 mediated 5′ end resection of telomere dsDNA.

### TRF2–RAP1 recruits BLM to protect telomere from engaging in HDR-mediated UT formation in U2OS cells

**Figure 7.**
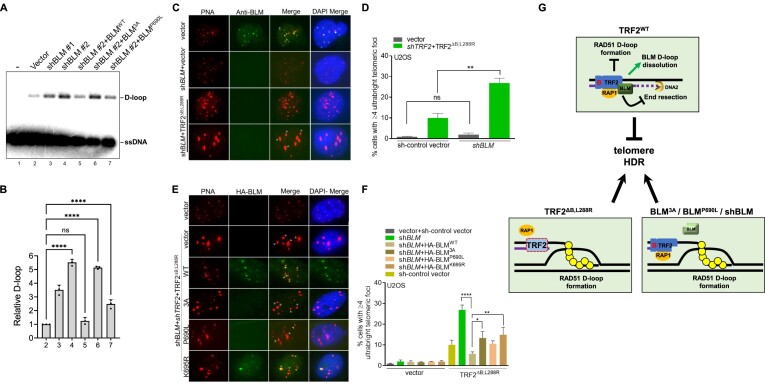
TRF2–RAP1-BLM protects telomeres from engaging in HDR-mediated D-loop and ultrabright telomere formation in U2OS cells. (**A**) Interaction of BLM with TRF2–RAP1 is required to prevent the accumulation of telomere D-loops. Telomere D-loop formation was examined using nuclear extracts isolated from U2OS cells expressing empty vector, shBLM#1, shBLM#2, and in BLM depleted cells expressing WT BLM or BLM mutants (3A and P690L). (**B**) The amount of D-loops formed relative to the empty vector from three independent experiments are quantified and shown as mean ± S.D. ANOVA test was performed in statistical analysis. ‘ns’ indicates non-significant (*P*= 0.7987); ****P < 0.0001. (**C**) Immunofluorescence-FISH analysis of BLM depleted U2OS cells expressing TRF2^ΔB; L288R^ revealed that BLM depletion promotes the generation of UTs. BLM was detected with anti-BLM antibody (green), telomeres visualized with the TelC-Cy3 (CCCTAA)_3_ PNA telomere probe (red) and DAPI stained nuclei (blue). Scale bars: 5 μm. Yellow arrows: co-localization of BLM on telomeres; white arrows: UTs. (**D**) Quantification of percentage of U2OS cells possessing UTs with or without treatment with BLM shRNA. Data represents the mean of two independent experiments ± SD from a minimum of 250 nucleus analyzed per experiment. Statistical analysis was performed by one-way ANOVA. non-significant: *P*= 0.93; **P= 0.0019. (**E**) Immunofluorescence-FISH analysis for HA-tagged WT BLM and indicated BLM mutants (green) co-localizing with telomeres (red) in U2OS cells expressing TRF2^ΔB; L288R^. Scale bars: 5 μm. Yellow arrows: co-localization of BLM with telomeres; white arrows: UTs. (**F**) Quantification of percent UTs observed in sh*BLM* treated U2OS cells reconstituted with sh*BLM* resistant WT BLM and the indicated BLM mutants in TRF2^ΔB; L288R^ expressing U2OS cells. Data represents the mean of two independent experiments ± SD from a minimum 200 nucleus analyzed per experiment. Statistical analysis was performed by ANOVA. *P= 0.0265; **P= 0.0066; ****P< 0.0001. (**G**) Model showing three distinct mechanisms of how TRF2–RAP1 prevents aberrant telomere HDR. First, RAP1 cooperates with TRF2 to inhibit telomere D-loop formation by blocking RAD51-mediated homology search on telomere dsDNA. Second, TRF2–RAP1 interaction with BLM promotes BLM-mediated telomeric D-loop unwinding. Third, TRF2–RAP1 represses BLM–DNA2-mediated telomere 5′-end DNA resection. In cells expressing TRF2^ΔB, L288R^ or BLM^3A^, uncontrolled BLM–DNA2-mediated 5′ end resection of telomere dsDNA generates extensive ssDNA, enabling RAD51 nucleoprotein filament assembly and invasion into telomere dsDNA, forming telomere D-loops, resulting in massive telomere HDR and formation of UTs.

We next asked whether UT formation in U2OS cells is dependent on BLM’s ability to interact with TRF2. Nuclear extracts were isolated from U2OS cells expressing empty vector, two different shRNAs targeting BLM (shBLM#1 and shBLM#2), and shBLM#2-treated cells reconstituted with either shRNA resistant WT BLM, BLM^3A^ or BLM^P690L^ mutants. We performed D-loop assays using reconstituted NEs and discovered that BLM depletion resulted in increased telomere D-loop formation (Figure 7A, lanes 3 and 4; Figure 7B). Reconstitution with WT BLM reduced telomere D-loop levels to those observed in NEs treated vector control (Figure 7A, compare lanes 2 and 5). In contrast, reconstitution with either BLM^3A^ or BLM^P690L^ mutants was unable to fully repress D-loop formation (Figure 7A, lanes 6 and 7). These results support our biochemical data that robust interaction between BLM and TRF2 is required for BLM to repress telomere D-loops in U2OS cells.

We next asked whether BLM interaction with TRF2 is required to repress UT formation in U2OS cells. U2OS cells were infected with shControl-vector or shBLM and reconstituted with either vector DNA or TRF2^ΔB, L288R^. As reported previously (27,30,31), endogenous BLM foci were readily detected on telomeres (Figures 7C, Supplementary Figure S7B). Depletion of BLM resulted in a significant increase in UT detected in cells expressing TRF2^ΔB, L288R^ (Figures 7C, D), suggesting that BLM represses UT formation. In addition, we reconstituted WT HA-BLM and HA-BLM mutants 3A, P690L and K695R in U2OS cells in which endogenous BLM was depleted. We found that only WT HA-BLM and the HA-BLM^K695R^ mutant localized to telomeres (Figure 7C–F; Supplementary Figure S7A–D). These results indicate that TRF2–RAP1 recruits BLM to telomeres and that the TRF2-BLM interaction is critical for BLM’s localization to telomeres. We note that BLM knockdown by itself does not induce UT formation in cells expressing vector cDNA, reinforcing the notion that expression of TRF2^ΔB, L288R^ and BLM depletion are both required to form UTs. We found that reconstitution of WT BLM in shBLM-treated U2OS cells expressing TRF2^ΔB, L288R^ reduced UT formation by ∼3-fold (Figures 7E, F). In contrast, reconstitution with the BLM^3A^ and BLM^P690L^ mutants defective in TRF2 interaction, or with the helicase-dead BLM^K695R^ mutant (41), all failed to repress UT formation to levels observed for WT BLM (Figures 7E, F, Supplementary Figure S7A). These results support our biochemical data that BLM’s helicase activity and its ability to interact with TRF2 are both required to repress telomere HDR and UT formation in U2OS cells.

## Discussion

We have previously shown that components of the shelterin complex evolved to inhibit specific DNA damage response and repair pathways (10,13–15,55,56). For example, we have shown that the POT1 protein protects telomere ends from engaging in ATR-dependent A-NHEJ-mediated repair (55). As the main telomere protector, TRF2 prevents the activation of ATM-dependent signaling and C-NHEJ-mediated telomere repair that would otherwise result in end-to-end chromosome fusions and cell death (8,9,11). RAP1 represses HDR-mediated telomere recombination but is dispensable for NHEJ-mediated telomere repair (13). Neither TRF2 nor RAP1 alone fully protects telomeres. Inappropriate HDR in mammalian cells lacking both the TRF2^B^ and RAP1 results in catastrophic telomere loss and telomere-free fusions (14). The accumulation of RPA and RAD51 on telomere filaments prior to the formation of UTs in the mutant cells suggests that RAD51-mediated HDR is involved in the early steps of telomere clustering (15). Similar to classic HDR, RAD51 also forms D-loops on telomere DNA (24). BLM has been shown to unwind D-loops on both DSBs and telomeres (27,57,58). Previous studies suggest that TRF2 plays a role in RAD51-mediated telomere D-loop formation and BLM-mediated D-loop unwinding (24,27). However, it has remained unclear mechanistically how RAP1 cooperates with TRF2 to inhibit telomere HDR.

In this study, we show mechanistically how the TRF2–RAP1 complex prevents telomere HDR (Figure 7G). Using purified proteins, we first show that RAP1 cooperates with TRF2 to inhibit RAD51-mediated D-loop formation on telomeres. This functional synergy requires both TRF2^B^ and RAP1 binding to TRF2. Specifically, the TRF2–RAP1 heterodimer blocks RAD51 coated ssDNA filaments to capture telomere dsDNA but does not affect RAD51 filament stabilization on telomere ssDNA. Second, we identified critical amino acid residues F686, L688 and P690 in BLM that are required for interaction with the TRF2 TRFH domain. Mutating these amino acids disrupt binding to TRF2 but still preserve BLM helicase activity, resulting in increased formation of telomere D-loops and UTs in cells. These results reveal that the TRF2–RAP1 complex and TRF2–BLM interaction are both critical to promote unwinding of the telomere D-loop to inhibit telomere HDR. Finally, our data revealed that TRF2–RAP1 inhibits BLM–DNA2-mediated telomere end resection, an early step required to generate RAD51 coated ssDNA filaments. Our findings provide the molecular basis for elucidating how TRF2–RAP1 orchestrates RAP1, BLM and RAD51 to inhibit telomere HDR.

TRF2 inhibits RAD51-mediated telomere D-loop formation through its Myb domain. In contrast, TRF1, another Myb-domain containing telomere binding protein, does not inhibit this reaction (24). RAP1 modulates TRF2^B^ to impact on its ability to interact with branched DNA structures (17–19). TRF2 is also able to induce positive supercoiling on telomere dsDNA (26). In contrast, the RAD51 filament efficiently forms D-loops only on negatively supercoiled DNA (59). Our biochemical data reveal that TRF2–RAP1 coated telomere dsDNA fails to be captured by the RAD51-telomere ssDNA filament (Figure 2D). We postulate that in the presence of RAP1, the improved selectivity of TRF2^B^ for telomere dsDNA further increases TRF2′s ability to induce positive dsDNA supercoiling, generating a substrate that is not efficiently captured by the RAD51 ssDNA filament. In this scenario, both TRF2^B^ and RAP1–TRF2 interaction would be expected to prevent RAD51-ssDNA-mediated strand invasion. In agreement with this notion, our data revealed that TRF2^ΔB, L288R^ is defective in inhibiting RAD51-mediated telomere D-loop formation (Figure 7G). Since TRF2 only binds telomere dsDNA, it is not surprising that TRF2–RAP1 did not impact RAD51-mediated filament formation on telomere ssDNA (Figure 2B).

It has been shown previously that BLM can disassemble the RAD51 filament on non-telomere ssDNA(41). Whether BLM also has the activity to disrupt RAD51 filament on telomere ssDNA and whether TRF2–RAP1 can modulate BLM to remove RAD51 from telomere ssDNA, are potentially interesting questions to address. Finally, the role that the nuclease SLX4 plays in telomere D-loop formation remains to be explored. We have previously shown that the nuclease SLX4 is critical for UT formation (15). Telomere D-loops can be nucleolytically resolved by the nuclease SLX4, which counteracts BLM’s unwinding activity (60,61). Since the TRF2–RAP1 interaction enhances BLM-mediated unwinding of telomere D-loops, it would be interesting to determine if this interaction inhibits SLX4′s activity on telomere D-loops.

The BLM helicase contributes to telomere maintenance by impacting telomere HDR in ALT tumors and by suppressing the activation of DNA fragile sites (62–64). BLM participates in HDR by influencing DNA end resection and resolving double Holliday junctions (65). Our results and those of others suggest that BLM unwinds telomere DNA substrates, including dsDNA, G4s, D-loops and 3′-overhangs (Figures 3 and 5) (27,28,66,67). In this study, we provide biochemical and genetic data to show mechanistically how TRF2 enhances BLM helicase activity on telomeres. Both TRF2–RAP1 complex formation and TRF2-BLM interaction are required to unwind RAD51-generated telomere D-loops. Importantly, our cell biology data reveal that BLM depletion results in the accumulation of telomere D-loops and UTs in U2OS cells. BLM mutants with reduced ability to interact with TRF2, or the BLM helicase-dead mutant are also compromised to prevent UT formation (Figure 7C). These results suggest that both BLM’s TRF2-binding ability and its helicase activity are both important to protect telomeres from engaging in aberrant HDR.

Our data also reveal a novel role for the TRF2–RAP1 complex in modulating BLM–DNA2-mediated 5′ end resection on telomere dsDNA lagging strand. We show that the telomere end resection activity of BLM–DNA2 is inhibited by TRF2–RAP1. Since BLM’s ability to interact with TRF2 only partially contributes to this inhibition, our results suggest a direct effect of TRF2–RAP1 on DNA2. In support of this notion, DNA2 was found to interact with TRF2 and localize to telomeres (53). DNA2 deficiency leads to sister telomere associations and telomere loss and DNA2^+/−^ MEFs display shorter telomeres and chromosome bridges (53). DNA2 is overexpressed in cancer cells, and its overexpression in yeast results in telomere dysfunction (68,69). Our study suggests that the DNA end resection activity of BLM–DNA2 on telomeres is tightly regulated by TRF2–RAP1 to ensure the generation of a proper 3′-overhang. Given that extensive 5′-end resection would generate long 3′-ssDNA overhangs that serves as a substrate for RAD51 mediated HDR, the inhibition of BLM–DNA2 end resection activity by TRF2–RAP1 provides another mechanism to prevent aberrant HDR on telomeres (Figure 7G). A recent report revealed that BLM promotes ALT by generating telomeric 5′ flaps during lagging strand synthesis to initiate telomere replication-associated damage response. Furthermore, DNA2 deficiency increased the BLM-dependent 5′ flap substrate (31). It will be of interest to test whether TRF2–RAP1 plays any role on the BLM–DNA2 activity on ALT telomeres.

## Supplementary Material

gkae642_Supplemental_File

## Data Availability

All data are incorporated into the article and its online supplementary material.
