# Supplementary material for: TRF2–RAP1 represses RAD51-dependent homology-directed telomere repair by promoting BLM-mediated D-loop unwinding and inhibiting BLM–DNA2-dependent 5′-end resection
Source: Nucleic Acids Res. 2024 Jul 31;52(16):9695–709. doi: 10.1093/nar/gkae642 (PMC11381343; doi:10.1093/nar/gkae642)
Supplement: gkae642_Supplemental_File [file gkae642_supplemental_file.pdf]

Supplementary data

TRF2-RAP1 represses RAD51-dependent homology-directed telomere repair by promoting BLM-mediated D-loop unwinding and inhibiting BLM-DNA2-dependent 5'-end resection

Fengshan Liang, Rekha Rai, Tori Sodeinde and Sandy Chang

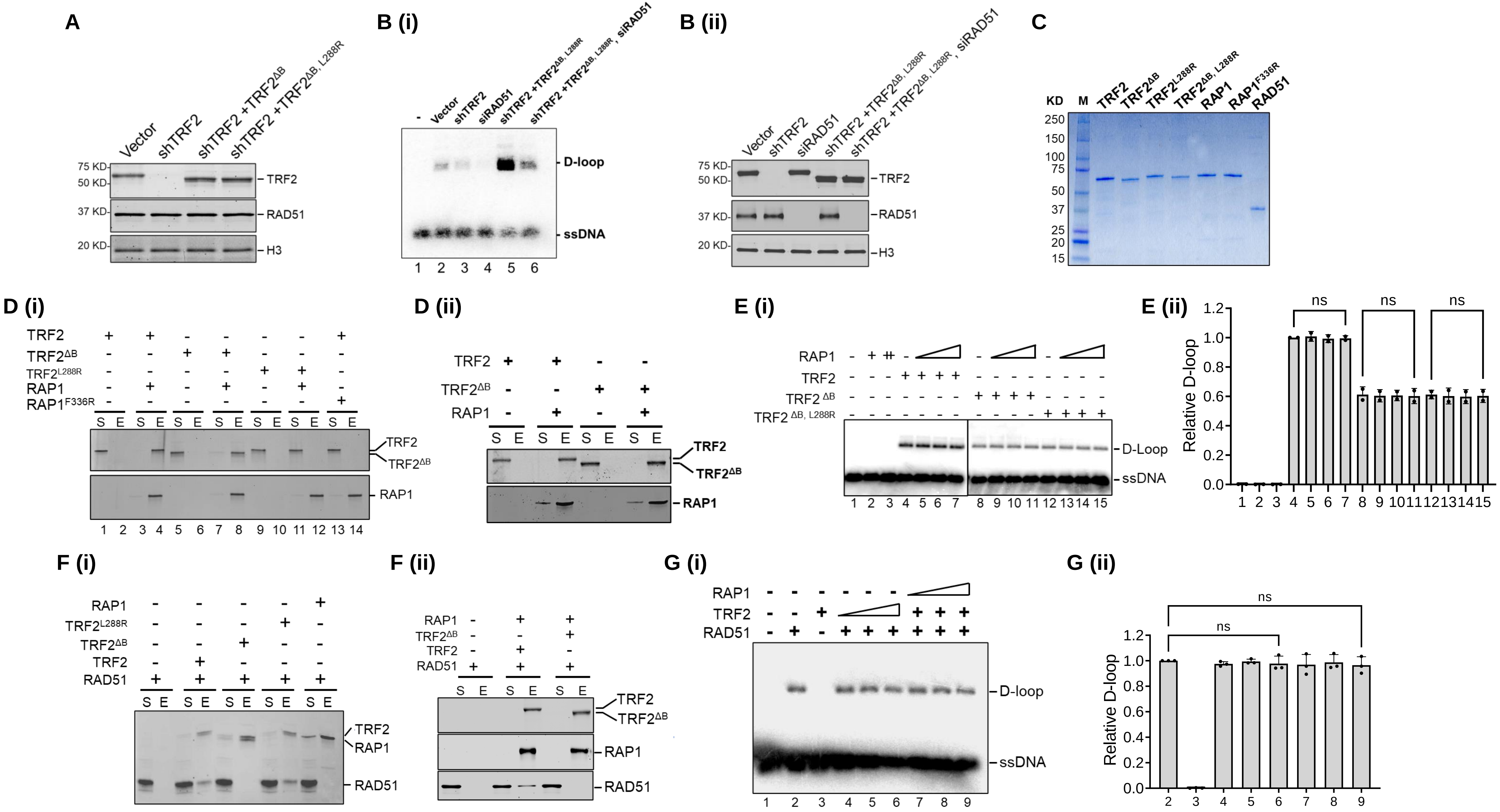
A
Vector
shTRF2
shTRF2 +TRF2ΔB
shTRF2 +TRF2ΔB, L288R
75 KD
50 KD
TRF2
37 KD
RAD51
20 KD
H3
B (i)
-
Vector
shTRF2
siRAD51
shTRF2 +TRF2ΔB, L288R
shTRF2 +TRF2ΔB, L288R, siRAD51
D-loop
ssDNA
1 2 3 4 5 6
B (ii)
Vector
shTRF2
siRAD51
shTRF2 +TRF2ΔB, L288R
shTRF2 +TRF2ΔB, L288R, siRAD51
75 KD
50 KD
TRF2
37 KD
RAD51
20 KD
H3
C
KD
M
TRF2
TRF2ΔB
TRF2L288R
TRF2ΔB, L288R
RAP1
RAP1F336R
RAD51
250
150
100
75
50
37
25
20
15
D (i)
TRF2
TRF2ΔB
TRF2L288R
RAP1
RAP1F336R
S E
TRF2
TRF2ΔB
RAP1
1 2 3 4 5 6 7 8 9 10 11 12 13 14
D (ii)
TRF2
TRF2ΔB
RAP1
S E
TRF2
TRF2ΔB
RAP1
E (i)
RAP1
TRF2
TRF2 ΔB
TRF2 ΔB, L288R
D-Loop
ssDNA
1 2 3 4 5 6 7 8 9 10 11 12 13 14 15
E (ii)
ns
Relative D-loop
1.2
1.0
0.8
0.6
0.4
0.2
0.0
1 2 3 4 5 6 7 8 9 10 11 12 13 14 15
F (i)
RAP1
TRF2L288R
TRF2ΔB
TRF2
RAD51
S E
TRF2
RAP1
RAD51
F (ii)
RAP1
TRF2ΔB
TRF2
RAD51
S E
TRF2
TRF2ΔB
RAP1
RAD51
G (i)
RAP1
TRF2
RAD51
D-loop
ssDNA
1 2 3 4 5 6 7 8 9
G (ii)
ns
Relative D-loop
1.2
1.0
0.8
0.6
0.4
0.2
0.0
2 3 4 5 6 7 8 9

Supplementary Figure 1. Protein expression, purification and characterization.

A. Protein expression levels. Nuclear extract from U2OS cells expressing empty vector, shTRF2, and shTRF2-depleted cells expressing TRF2$^{\Delta B}$ or TRF2$^{\Delta B,\ L288R}$ mutants were examined for TRF2 and RAD51 expression by Western blotting with anti-TRF2 and anti-RAD51 antibodies. The expression of Histone H3 was used as loading control.

B(i). RAD51 is required for telomere D-loop formation in cells expressing TRF2 $^{\Delta B,\ L288R}$. Nuclear extract from the indicated U2OS cells were used to test telomere D-loop formation as shown in Figure 1B. Knockdown of RAD51 was achieved using siRNA against the RAD51 target sequence 5′-CUAAUCAGGUGGUAGCUCAUU-3′.

B(ii). Protein expression in reconstituted cell lines. Nuclear extract from indicated U2OS cells were used to test TRF2 and RAD51 expression by Western blotting with anti-TRF2 and anti-RAD51 antibodies. The expression of Histone H3 was used as loading control.

C. Coomassie Blue-stained SDS-PAGE gel showing purified human WT or mutant TRF2, RAP1, RAD51 proteins. M: molecular weight markers.

D(i). Interaction between TRF2 and RAP1 proteins. His-tagged WT TRF2, TRF2$^{\Delta B}$, or TRF2 ^{L288R} proteins were incubated with Flag-tagged WT RAP1 or the RAP1^{F336R} protein. Protein complexes were captured on M2 anti-Flag affinity resin and separated by SDS-PAGE. The supernatant (S), and eluate (E) fractions were detected by Western blotting with antibodies against TRF2 or Flag (for RAP1), as indicated.

D(ii). Interaction between TRF2 and RAP1 proteins remain stable at high salt (300 mM KCl). His-tagged WT TRF2 or TRF2$^{\Delta B}$ proteins were incubated with Flag-tagged RAP1. Protein complexes were captured on M2 anti-Flag affinity resin and the different fractions were separated by SDS-PAGE. The supernatant (S), and eluate (E) fractions were detected by Western blotting with antibodies against TRF2 or Flag (for RAP1), as indicated.

E(i). RAP1 does not affect TRF2 telomere D-loop activity. Left panel: RAP1 does not affect TRF2-mediated telomere D-loop formation. TRF2, TRF2$^{\Delta B}$, or TRF2$^{\Delta B,\ L288R}$ (400 nM) and RAP1 (200, 400 and 800 nM) proteins were tested for their ability to form telomere D-loops.

E(ii). D-loop formation relative to wild type TRF2 (lane 4) from two independent experiments were analyzed. Mean values ± S.D. were plotted. Data were evaluated by one-way ANOVA analysis. "ns" indicates no significant differences ($p>0.05$).

F(i). TRF2 protein binds RAD51 through its basic domain. RAP1 protein does not interact with RAD51. His-tagged TRF2 wild type, $TRF2^{\Delta B}$, or $TRF2^{L288R}$ mutants or RAP1 were incubated with untagged RAD51 and tested for interaction via the His tag on TRF2 or RAP1. The eluates from Ni Sepharose 6 Fast Flow resin were analyzed by SDS-PAGE and Coomassie Blue staining. S: supernatant containing unbound proteins; E: SDS eluate of the affinity resin.

F(ii). $TRF2^{\Delta B}$-RAP1 does not regain the ability to interact with RAD51. His-tagged WT TRF2 or $TRF2^{\Delta B}$ proteins were incubated with Flag-tagged RAP1 and untagged RAD51. Protein complexes were captured on M2 anti-Flag affinity resin. The supernatant (S) and eluate (E) fractions were analyzed by SDS-PAGE and detected by Western blotting with antibodies against TRF2 or Flag (for RAP1), and RAD51.

G(i). TRF2-RAP1 does not affect RAD51-mediated D-loop formation on non-telomere DNA. RAD51(800 nM), TRF2 and RAP1 (200, 400, 800 nM) were tested for the ability to form non-telomere D-loops.

G(ii). The D-loops relative to RAD51 alone (lane 2) were analyzed as in Figures 1E and 1F. The data from three independent experiments are shown as the mean ± S.D. Statistical evaluation was performed by unpaired t test analysis. ns, non-significant ($p>0.05$).

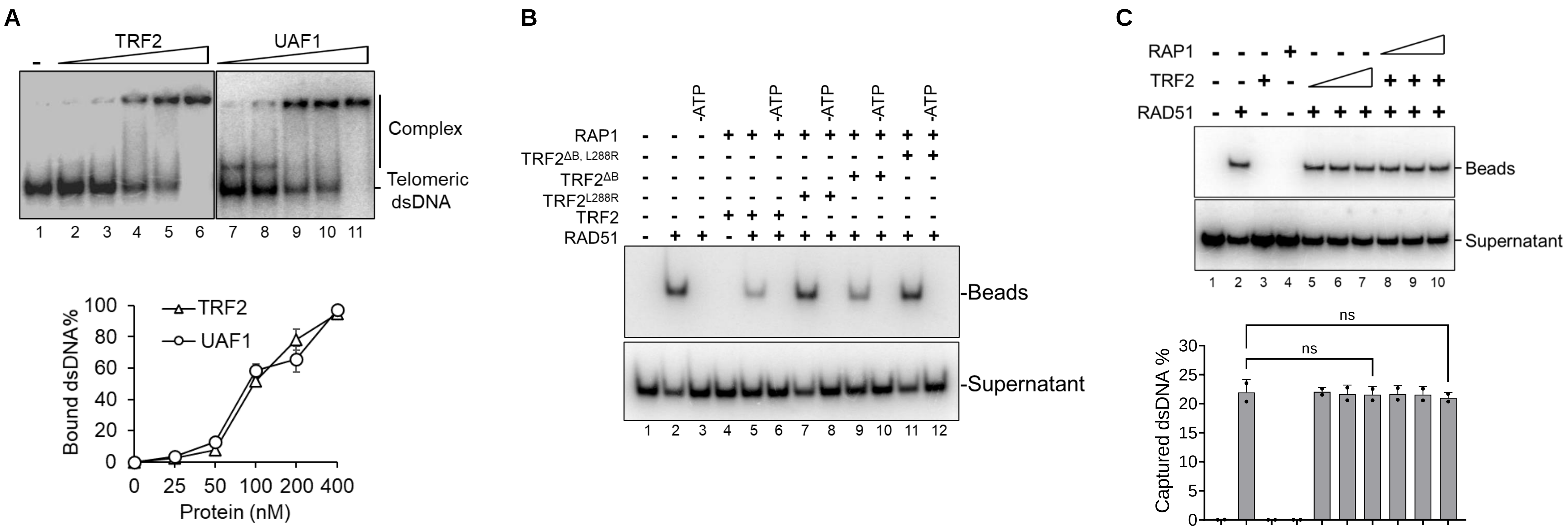
A
TRF2
UAF1
Complex
Telomeric dsDNA
1 2 3 4 5 6 7 8 9 10 11
TRF2
UAF1
Bound dsDNA%
Protein (nM)
0 25 50 100 200 400
B
-ATP
RAP1
TRF2ΔB, L288R
TRF2ΔB
TRF2L288R
TRF2
RAD51
-Beads
-Supernatant
1 2 3 4 5 6 7 8 9 10 11 12
C
RAP1
TRF2
RAD51
Beads
Supernatant
1 2 3 4 5 6 7 8 9 10
ns
ns
Captured dsDNA %

Supplementary Figure 2. The TRF2-RAP1 complex inhibits RAD51-mediated telomere duplex capture in an ATP-dependent manner.

A. Both TRF2 and UAF1 bind telomere dsDNA. Proteins of the indicated concentration were incubated with radiolabeled telomere dsDNA and the mobility shift of the DNA-protein complexes were analyzed by a 10% native polyacrylamide gel. Bottom: quantification of amount of bound dsDNA in a protein concentration dependent manner. The error bars represent mean values ± S.D. of data from three independent experiments.

B. The TRF2-RAP1 complex inhibits RAD51-mediated telomere duplex capture in an ATP-dependent manner. TRF2, TRF2$^{\Delta B}$, TRF2^{L288R} or TRF2$^{\Delta B, L288R}$ and RAP1 proteins (200 nM) were incubated with RAD51 coated telomere ssDNA filaments, and their ability to capture telomere dsDNA was analyzed as in Figure 2E. ATP was omitted from the reaction in lanes 3, 6, 8, 10 and 12.

C. TRF2-RAP1 does not affect RAD51-ssDNA mediated capture of non-telomere dsDNA. TRF2 either singly or in combination with RAP1 was incubated with RAD51-ssDNA filament generated on non-telomere ssDNA, and the ability to capture ^{32}P-labled non-telomere dsDNA was examined. The percentages of captured dsDNA from three independent experiments are plotted and analyzed. Statistical evaluation was performed by unpaired t test analysis. Ns: non-significant ($p>0.05$).

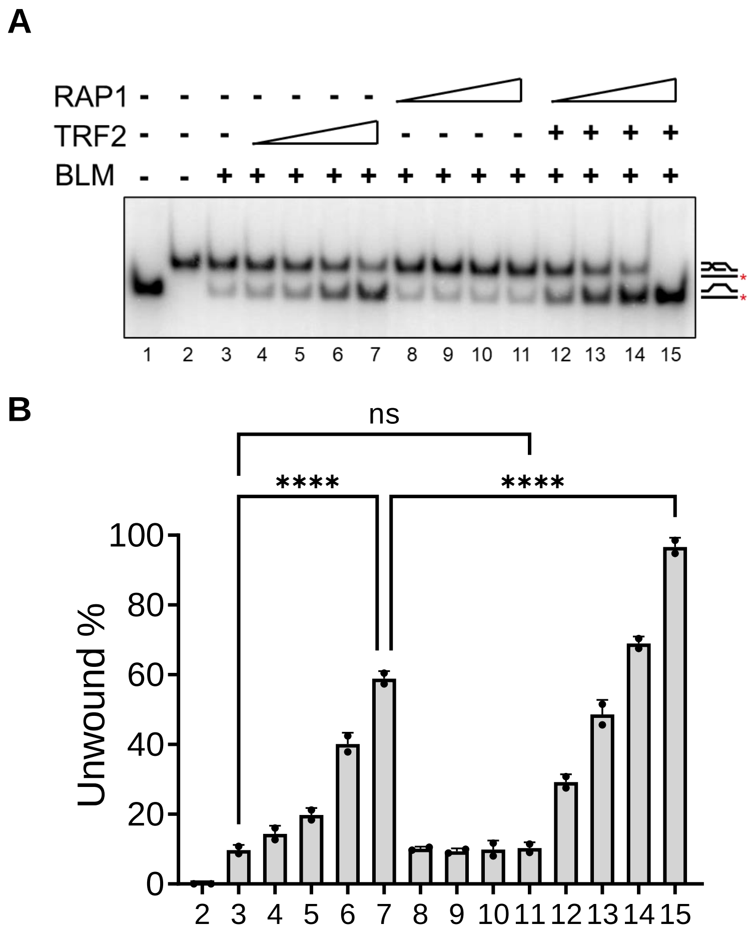
A
RAP1
TRF2
BLM
1 2 3 4 5 6 7 8 9 10 11 12 13 14 15
B
ns
****
****
Unwound %
0
20
40
60
80
100
2 3 4 5 6 7 8 9 10 11 12 13 14 15

Supplementary Figure 3. TRF2-RAP1 promotes BLM-mediated telomere D-loop unwinding.

A. TRF2, but not RAP1, promotes BLM-mediated telomere D-loop unwinding. The addition of RAP1 to TRF2 further enhanced telomere D-loop unwinding by BLM. The effect of TRF2 individually or in combination with RAP1 (10, 20, 40, 80 nM) on BLM ‘s ability to unwind telomere D-loops was quantified and analyzed.

B. Statistical difference was evaluated via ANOVA test. Ns: non-significant ($p>0.05$); ****: $p<0.0001$.

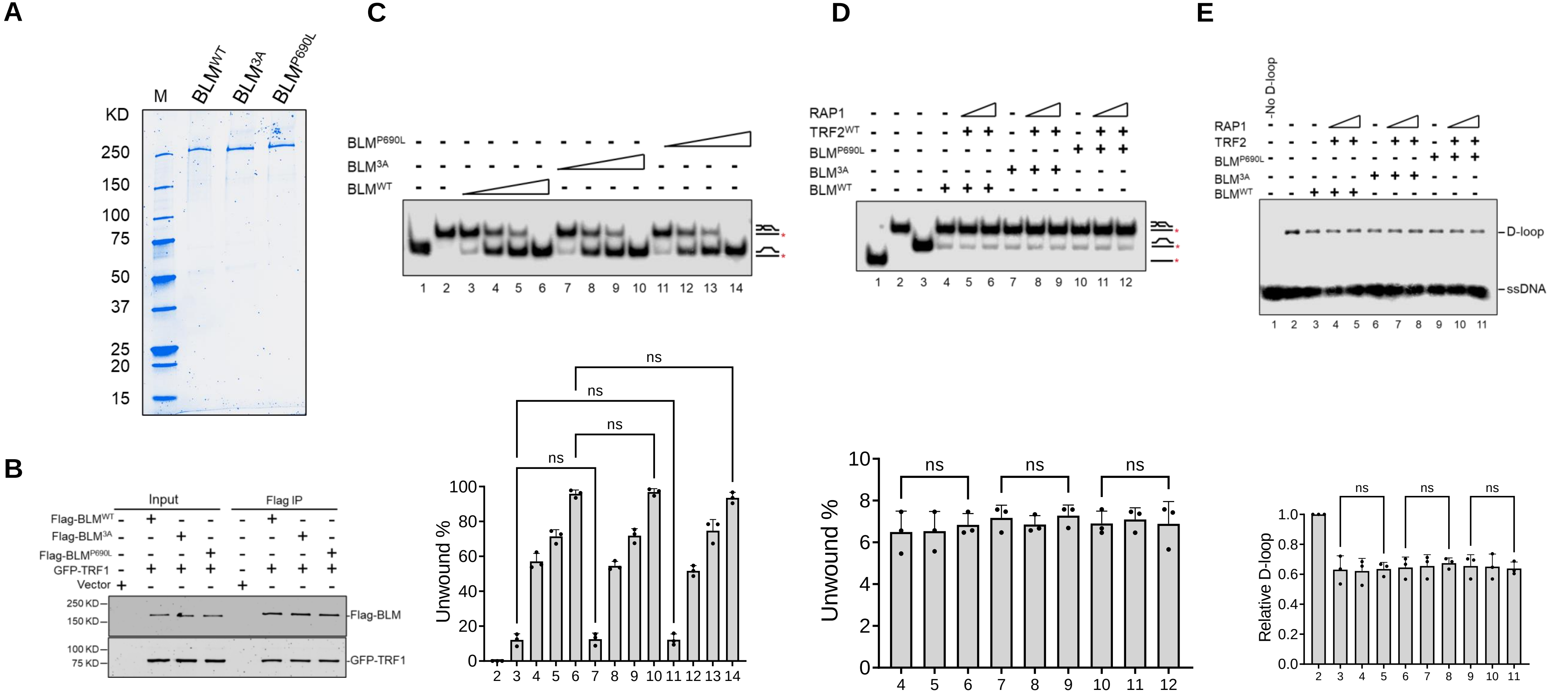
A
B
C
D
E
M
BLMWT
BLM3A
BLMP690L
KD
250
150
100
75
50
37
25
20
15
Input
Flag IP
Flag-BLMWT
Flag-BLM3A
Flag-BLMP690L
GFP-TRF1
Vector
250 KD
150 KD
100 KD
75 KD
Flag-BLM
GFP-TRF1
RAP1
TRF2WT
TRF2
-No D-loop
D-loop
ssDNA
Unwound %
Relative D-loop
ns

Supplementary Figure 4. BLM protein purification and examination of DNA helicase activity.

A. Purified WT BLM and BLM mutants 3A, P690L are detected by Coomassie Blue-stained SDS-PAGE. M: molecular weight markers.

B. Interaction of Flag-tagged WT BLM or BLM mutants 3A or P690L with GFP-tagged TRF1 detected by co-immunoprecipitation reveal robust interaction between BLM and TRF1. Proteins were separated and detected by Western blotting using anti-Flag (for BLM) and anti-GFP (for TRF1) antibodies.

C. Top: WT BLM and BLM mutants (3A or P690L) all possess robust DNA helicase activity. BLM proteins (20, 40, 60, 80 nM) were incubated with telomere D-loops and BLM unwinding activity was examined. ^{32}P-labled dsDNA without the invading ssDNA strand and labeled D-loops were loaded as molecular weight markers (lanes 1 and 2).

Bottom: The percentages of unwound D-loops from three independent experiments were shown as mean ± S.D. in bottom panel. Statistical difference was evaluated via ANOVA test. ns: non-significant ($p>0.05$).

D and E. Top: the effect of TRF2-RAP1 on BLM-mediated unwinding of non-telomere oligo-generated D-loops (**D**) or RAD51-generated D-loops (**E**) were examined. Bottom: the percentage of unwound oligo-based D-loops (**D**) or Rad51 D-loops (E) are shown as mean ± S.D. from three independent experiments. Statistical evaluation was performed by unpaired t test analysis. Ns: non-significant ($p>0.05$).

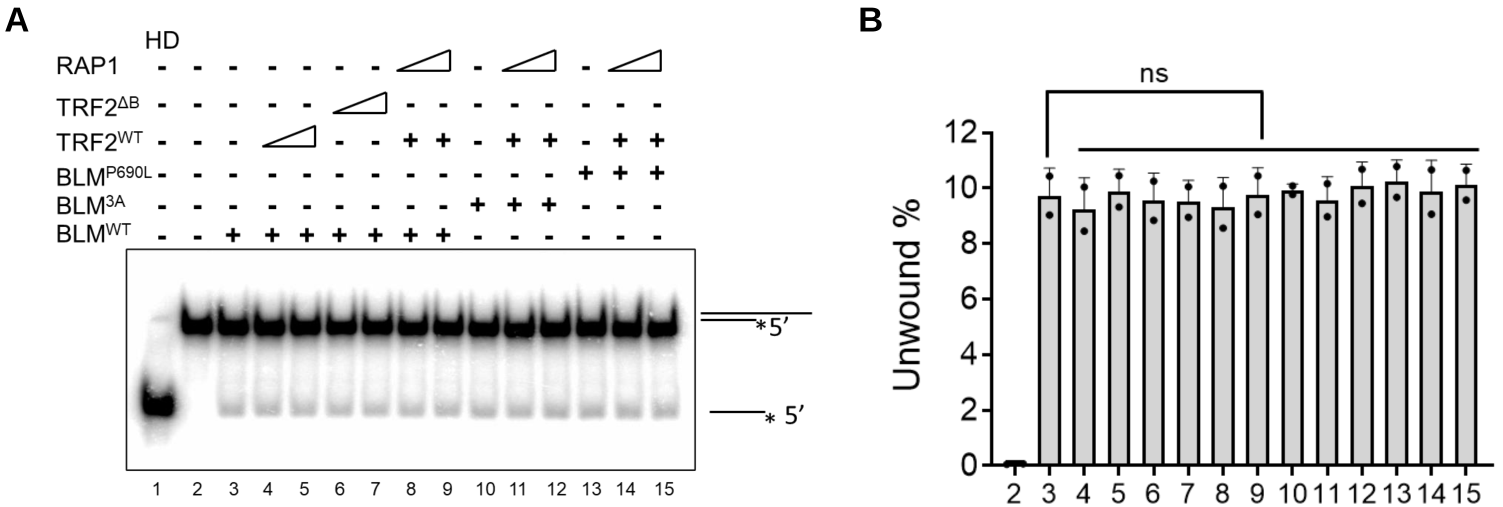
A
HD
RAP1
TRF2ΔB
TRF2WT
BLMP690L
BLM3A
BLMWT
*5′
* 5′
1 2 3 4 5 6 7 8 9 10 11 12 13 14 15
B
ns
Unwound %
0 2 4 6 8 10 12
2 3 4 5 6 7 8 9 10 11 12 13 14 15

Supplementary Figure 5. TRF2-RAP1 does not affect BLM-mediated unwinding of non-telomere 3'-overhangs.

A. TRF2 individually or in combination with RAP1 was incubated with non-telomere 3'-overhang DNA together with WT BLM or BLM mutants 3A and P690L to detect unwinding of the 3'-overhang. Heat denatured (HD) DNA and 3'-overhang DNA were loaded in lane 1 and 2 as molecular weight markers.

B. Quantification of unwinding of the non-telomere 3'-overhang DNA shown in Figure S5A. The percentage of unwound non-telomere 3'-overhangs were shown as mean ± S.D. from two independent experiments. "ns" denotes non-significant difference ($p>0.05$) between reactions by unpaired t test statistical analysis.

Figure S6- Liang *et al*

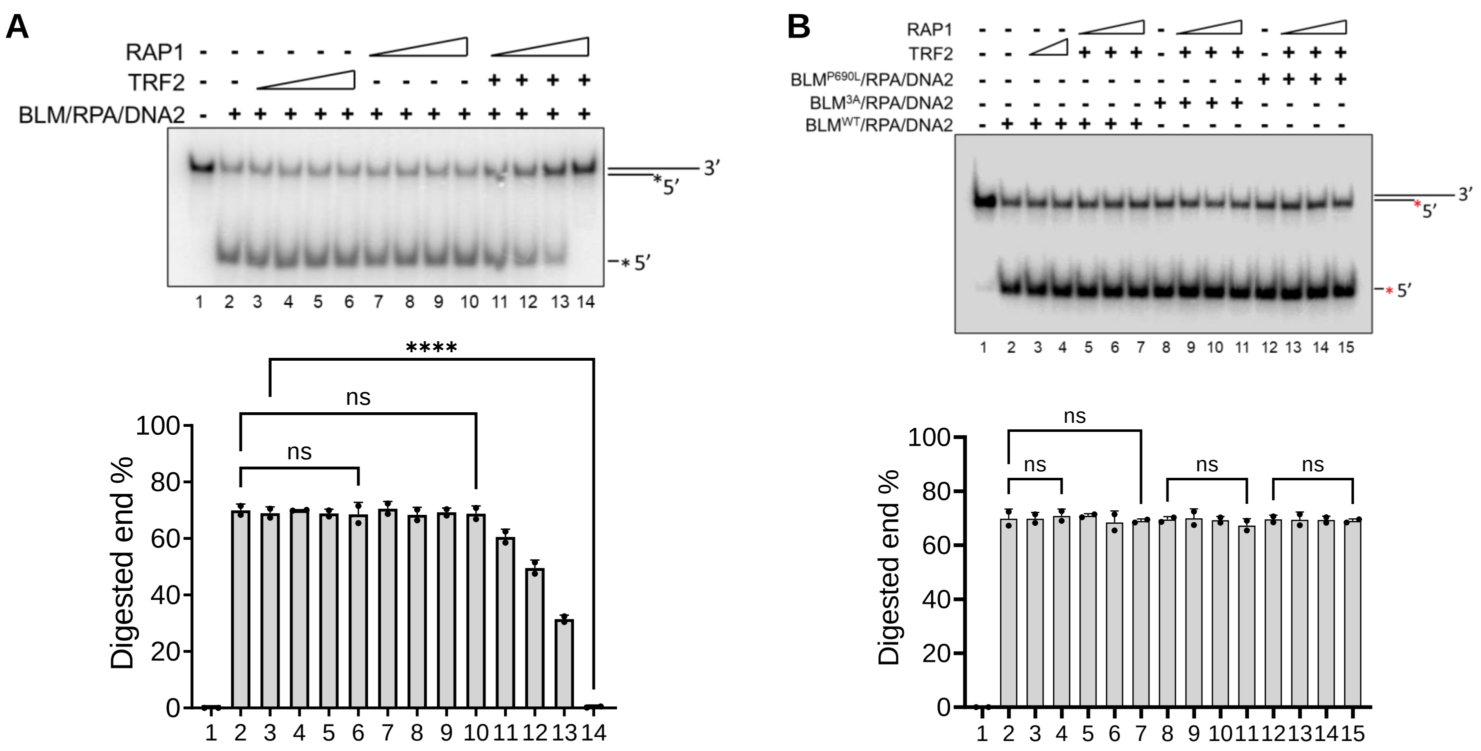

Supplementary Figure 6. The TRF2-RAP1 complex inhibits 5' end resection of telomere DNA but not non-telomere DNA.

A. The TRF2-RAP1 complex but not TRF2 or RAP1 individually inhibits end resection of the telomere 5'-DNA. The effect of TRF2 individually or in combination with RAP1 (20, 40, 80, 120 nM) was examined.

Bottom: the percentages of digested 5'-ends are shown as mean ± S.D. from two independent experiments. "ns" indicates non-significant ($p>0.05$); ****: $p<0.0001$.

B. TRF2-RAP1 does not affect the end resection activity of BLM/RPA/DNA2 on non-telomere 3'-overhangs. TRF2 (40, 80 nM), RAP1 (40, 80, 120 nM) and BLM/RPA/DNA2 were examined as indicated.

Bottom: the percentages of digested 5'-end were quantified and the data from three independent experiments were plotted as mean ± S.D. Statistical analysis by ANOVA indicates that there is no-significant difference (ns) between samples.

Figure S7- Liang *et al*

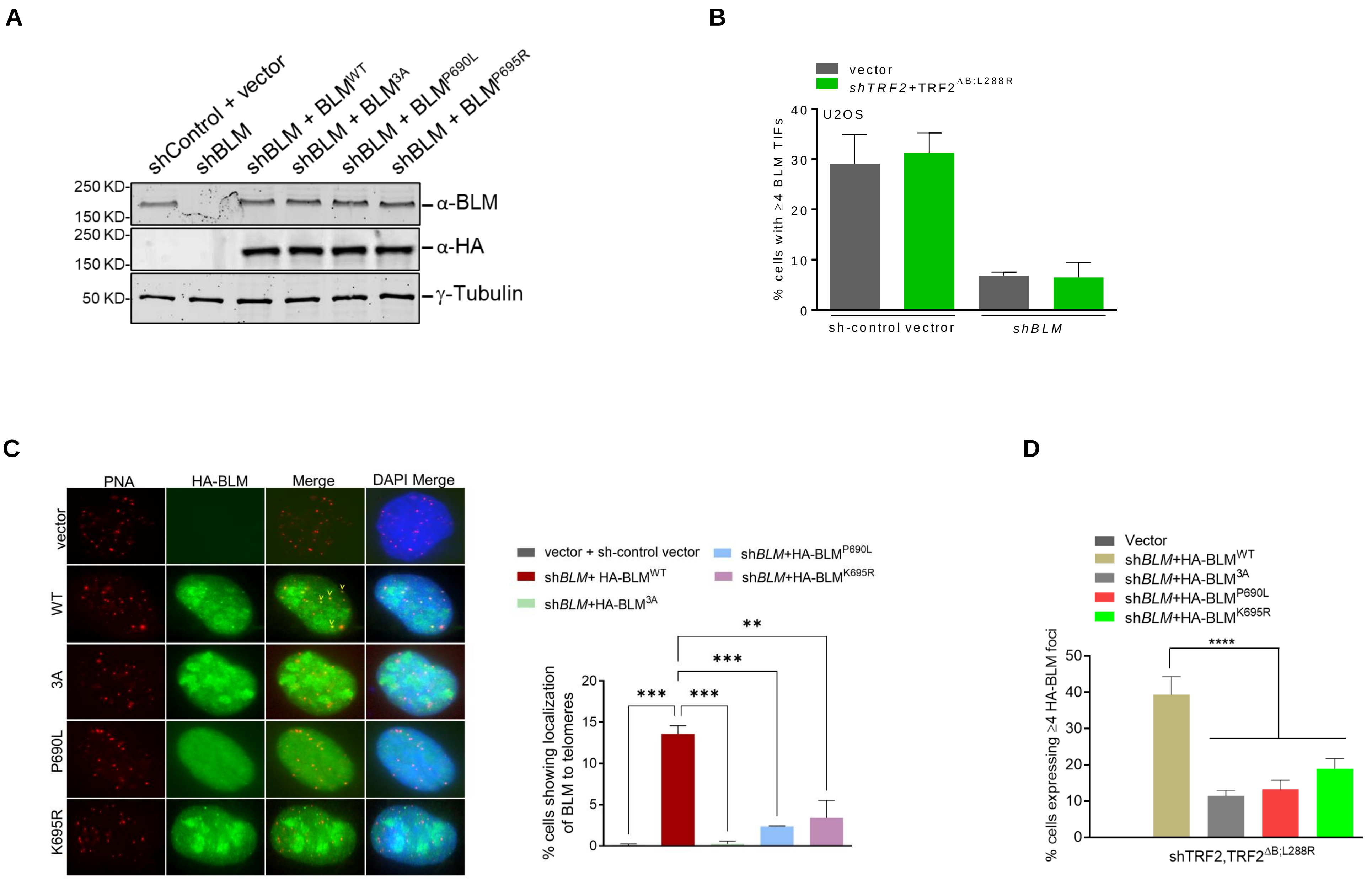

Supplementary Figure 7. BLM localizes to telomeres of U2OS cells to repress UT formation.
A. U2OS cells were infected with shBLM targeting the *Blm* 3'-UTR and reconstituted with shBLM resistant HA-tagged cDNAs encoding either WT BLM or mutant BLM. Protein levels in cells were quantified by Western blotting with anti-BLM and anti-HA antibodies.
B. Quantification of percent of cells displaying BLM localization on telomere shown in Figure 7C. The data are shown as mean ± S.D. from two independent experiments.
C. WT BLM localizes to telomeres. Left: U2OS cells were infected with sh-control vector or shBLM targeting the endogenous *Blm* 3'-UTR and reconstituted with shBLM-resistant HA-tagged cDNAs encoding either WT BLM or mutant BLM. Immunofluorescence-FISH analysis revealed that only WT BLM was able to efficiently localize to telomeres (arrows). BLM was detected with anti-HA antibody (green), telomeres visualized with the TelC-Cy3 $(CCCTAA)_3$ PNA telomere probe (red) and DAPI stained nuclei (blue). Yellow arrows: co-localization of HA-BLM on telomeres. Right: Quantification of percentage of U2OS cells with HA-BLM localized to telomeres. Data represents the mean of two independent experiments ±SD from a minimum of 250 nucleus analyzed per experiment. Statistical analysis was performed by one-way ANOVA. *: p<0.05; **: p<0.01; ***: p<0.001.
D. BLM mutants show telomere localization defects in U2OS cells expressing shTRF2 +$TRF2^{\Delta B; L288R}$. Quantification of percent cells displaying BLM localization in Figure 7E are shown as mean ± S.D. from two independent experiments. Statistical analysis was performed by one-way ANOVA. *:p=0.017; **: p=0.0024 and 0.0088; ****: p<0.0001.

Table S1 Oligonucleotides used in this study

Name	Sequence (5'-3')	Assay
T100	TTAGGGTTAGGGTTAGGGTTAGGGTTAGGGTTAGGGTTAGGGTTAG GGTTAGGGTTAGGGTTAGGGTTAGGGTTAGGGTTAGGGTTAGGGTT AGGGTTAG	Telomere D-loop assay
90mer	AAATCAATCTAAAGTATATATGAGTAAACTTGGTCTGACAGTTACC AATGCTTAATCAGTGAGGCACCTATCTCAGCGATCTGTCTATTT	Non-telomere D-loop assay
TD1	AGCTCCTAGGGTTACAAGCTTCACTAGGGTTGTCCAGTCACAGTCA GAGTCACAGTCCTACACATGTAGGGTTGATCAGC	Telomere D-loop generation
TD2	GCTGATCAACCCTACATGTGTAGGTAACCCTAACCCTAACCCTAAG GACAACCCTAGTGAAGCTTGTAACCCTAGGAGCT	Telomere D-loop generation
TD3	AAAAAAAAAAAAAAATTAGGGTTAGGGTTAGGGTTA	Telomere D-loop generation
NonTD1	CATATTTAAAACATGTTGGAAGGCTCGATGCATGCTGATAGCCTAC TAGTGCTGCTGGCTTTCAAATGACCTCTTATCAA	Non-telomere D-loop generation
NonTD2	TTGATAAGAGGTCATTTGAAAGCCGGCTTAGAGCTTAATTGCTGAG CATGCATCGAGCCTTCCAACATGTTTTAAATATG	Non-telomere D-loop generation
NonTD3	CTGCTACGATGCTAG TCAGCAATTAAGCTCTAAGCC	Non-telomere D-loop generation
T3O1	AGCTGAGCATGTCCAGACATGTCCTAGGGTTAGGGTTAGGGTTAGG GTTAGGGTTAGGGTTAGGGTTAGGGTTAGGG	Telomere 3'-overhang
T3O2	TAACCCTAACCCTAGGACATGTCTGGACATGCTCAGCT	Telomere 3'-overhang
NonT3O1	AGCTGAGCATGTCCAGACATGTCCGTGAGTGTGAGTGTGAGTGTGA GTGTGAGTGTGAGTGTGAGTGTGAGTGTGAG	Non-telomere 3'-overhang
NonT3O2	ACACTCATACTCACGGACATGTCTGGACATGCTCAGCT	Non-telomere 3'-overhang
NonTDC1	TTATATCCTTTACTTTGAATTCTATGTTTAACCTTTTACTTATTTTGT ATTAGCCGGATCCTTATTTCAATTATGTTCAT	Non-telomere DNA capture
NonTDC2	ATGAACATAATTGAAATAAGGATCCGGCTAATACAAAATAAGTAA AAGGTTAAACATAGAATTCAAAGTAAAGGATATAA	Non-telomere DNA capture
